# Structural basis for substrate recognition and processive cleavage mechanisms of the trimeric exonuclease PhoExo I

**DOI:** 10.1093/nar/gkv654

**Published:** 2015-07-02

**Authors:** Ken-ichi Miyazono, Sonoko Ishino, Kanae Tsutsumi, Tomoko Ito, Yoshizumi Ishino, Masaru Tanokura

**Affiliations:** 1 Department of Applied Biological Chemistry, Graduate School of Agricultural and Life Sciences, The University of Tokyo, Tokyo, Japan; 2 Department of Bioscience and Biotechnology, Graduate School of Bioresource and Bioenvironmental Sciences, and Faculty of Agriculture, Kyushu University, Fukuoka, Japan

## Abstract

Nucleases play important roles in nucleic acid processes, such as replication, repair and recombination. Recently, we identified a novel single-strand specific 3′-5′ exonuclease, PfuExo I, from the hyperthermophilic archaeon *Pyrococcus furiosus*, which may be involved in the Thermococcales-specific DNA repair system. PfuExo I forms a trimer and cleaves single-stranded DNA at every two nucleotides. Here, we report the structural basis for the cleavage mechanism of this novel exonuclease family. A structural analysis of PhoExo I, the homologous enzyme from *P. horikoshii* OT3, showed that PhoExo I utilizes an RNase H-like active site and possesses a 3′-OH recognition site ∼9 Å away from the active site, which enables cleavage at every two nucleotides. Analyses of the heterotrimeric and monomeric PhoExo I activities showed that trimerization is indispensable for its processive cleavage mechanism, but only one active site of the trimer is required.

## INTRODUCTION

The DNA of every organism is constantly threatened by exogenous and endogenous DNA-damaging agents that induce base and sugar modifications, single- and double-strand breaks in DNA. If DNA lesions are left unrepaired, they can cause genome instability or cell death. To maintain genomic integrity, cells have evolved sophisticated DNA repair systems (1,2), including homologous recombination repair (HR) (3), mismatch repair (4), nucleotide excision repair (5) and base excision repair (6). Because Archaea, the third domain of life, have DNA repair proteins similar to those of Eukarya, structural and biochemical characterizations of archaeal homologs have revealed a number of important insights into the structures and functions of eukaryotic DNA repair proteins (7–10). In addition to the similarity between the DNA repair proteins of Archaea and Eukarya, it has been suggested that hyperthermophilic archaea must have extremely efficient and specialized DNA repair systems to survive in an inhospitable environment because they are always threatened by high temperatures that accelerate spontaneous mutations in DNA and by other DNA-damaging factors, such as ionizing radiation and chemical agents (11,12). *Pyrococcus furiosus*, one of the most studied hyperthermophilic archaea, grows optimally at over 100°C under anaerobic conditions (13). *P. furiosus* possesses an extremely active DNA repair mechanism to survive at high temperatures; the chromosome fragmentation of *P. furiosus* cells caused by exposure to ionizing radiation was fully repaired upon incubation at 95°C (14). However, details about the components and mechanism of the hyperthermophilic archaea-specific DNA repair system remain unknown.

Deoxyribonucleases (DNases) are one of the components of most DNA repair systems. DNases can execute or initiate the removal of damaged DNAs to ensure genomic integrity and promote cell survival (15). DNases are classified into two general groups according to their cleavage pattern: (i) endonucleases that hydrolyse internal phosphodiester bonds without the requirement of a free end and (ii) exonucleases that hydrolyse phosphodiester bonds from either the 5′ or 3′ end. Among the exonucleases, single-strand-specific 3′-5′ exonucleases participate in several repair processes within *Escherichia coli* and human cells (16). Although functionally similar exonucleases are expected to be involved in the DNA repair systems of *P. furiosus*, knowledge about the single-stranded DNA (ssDNA)-specific 3′-5′ exonucleases in *P. furiosus* is limited except for the DNA polymerase-associated proofreading exonuclease activity (17,18). In a previous study, we identified a novel single-strand-specific 3′-5′ exonuclease, PfuExo I, from *P. furiosus* (19). Homologous enzymes of PfuExo I are found only in the Thermococcales (Supplementary Figure S1), and their amino acid sequences show no similarity to any other protein with a known function. Although the precise cellular function of PfuExo I remains unclear, a report that the amount of PfuExo I mRNA increased after ionizing irradiation (20) may indicate that PfuExo I participates in a Thermococcales-specific DNA repair system. PfuExo I shows some characteristic biochemical features: it forms a homotrimer; it prefers poly-dT as a substrate; it cleaves ssDNA, but not double-stranded DNA (dsDNA), at every two nucleotides in the 3′ to 5′ direction, although most exonucleases cleave only one nucleotide at a time (19). A low-resolution (3.38 Å) crystal structure of PfuExo I has been reported as a hypothetical protein with unknown function (21). However, this report did not show any concrete function, and the structural basis for the substrate recognition and the DNA cleavage mechanisms of PfuExo I as a nuclease remained unclear.

Here, we report the crystal structures of PhoExo I, which shares 76% amino acid sequence identity with PfuExo I (22). In this study, we determined three substrate-free structures of PhoExo I and four structures of PhoExo I in complexes with ssDNAs. Based on these structures and on accompanying biochemical data, we have revealed the mechanism by which PhoExo I cleaves ssDNA at every two nucleotides from its 3′-end. In addition, the structures suggested that PhoExo I cleaves RNA in addition to DNA, and its RNase activity was experimentally confirmed. We also analysed the mechanism of processive DNA cleavage by PhoExo I. Although the chemistry of the nuclease reaction is well understood (15), knowledge about the processive cleavage mechanisms of exonucleases is limited (23,24). The results of the 3′-5′ exonuclease assay of the heterotrimeric and the monomeric PhoExo I revealed how the trimeric PhoExo I cleaves ssDNA in a processive manner.

## MATERIALS AND METHODS

### Expression and purification of PhoExo I

A gene fragment of PhoExo I (DDBJ accession no. AB935327) was amplified by PCR from genomic DNA of *P. horikoshii* OT3 and was cloned into the NdeI/BamHI site of the pET26b plasmid (Novagen). The constructed plasmid (pET26b-PhoExo I) was transformed into *E. coli* Rosetta (DE3) for protein expression. The transformants were cultivated at 37°C in LB medium containing 20 μg/ml kanamycin until the optical density at 600 nm of the medium reached 0.6. The expression of PhoExo I was induced by the addition of 0.1 mM (final concentration) isopropyl β-D-1-thiogalactopyranoside (IPTG). After cultivation at 18°C for 18 h, cells were harvested by centrifugation at 5000 x g for 10 min. The harvested cells were resuspended in 20 mM Tris HCl (pH 8.0) and 200 mM NaCl and were disrupted by sonication. After centrifugation at 40 000 × g for 30 min, the supernatant was incubated at 80°C for 30 min to denature the heat-labile *E. coli* proteins. After centrifugation at 40 000 × g for 30 min, ammonium sulfate was added to the supernatant until 80% saturation. The precipitant from the ammonium sulfate treatment was collected by centrifugation at 20 000 × g for 30 min and was dissolved in 20 mM Tris HCl (pH 8.0), 200 mM NaCl and 1.5 M ammonium sulfate. The protein solution was purified using a Resource PHE (GE Healthcare) column pre-equilibrated with 20 mM Tris HCl (pH 8.0) and 1.5 M ammonium sulfate, and it was eluted with a linear gradient of 1.5–0 M ammonium sulfate. PhoExo I was further purified using a Resource Q (GE Healthcare) column pre-equilibrated with 20 mM Tris HCl (pH 8.0) and was eluted with a linear gradient of 0–1 M NaCl. PhoExo I was also expressed in inclusion bodies in *E. coli*, solubilized by the high-pressure refolding method and purified with a Resource Q column as previously described (22).

For the expression of the Se-Met derivative of PhoExo I, the transformants were cultivated at 37°C in M9 medium supplemented with 0.5 mg/l thiamine, 0.4% glucose, 1 mM MgSO_4_ and 4.2 mg/l Fe_2_SO_4_ until the optical density at 600 nm of the medium reached 0.3. After that, 50 mg/l of Ile, Leu, Val and Se-Met as well as 100 mg/l of Lys, Phe and Thr was added to the medium. The expression of Se-Met-labelled PhoExo I was induced by the addition of 0.1 mM IPTG after the addition of the amino acids. After cultivation at 18°C for 18 h, cells were harvested by centrifugation at 5000 x g for 10 min. The Se-Met-labelled PhoExo I was purified by the same method as the native PhoExo I purification.

To obtain C-terminal 4×His-tagged PhoExo I (PhoExo I-CHis), the pET26b-PhoExo I plasmid was modified using the PrimeSTAR Mutagenesis Basal Kit (TaKaRa). The modified plasmid (pET26b-PhoExo I-CHis) was transformed into *E. coli* Rosetta (DE3) for protein expression. The overexpression of PhoExo I-CHis was performed using the same method used for the native PhoExo I overexpression. The harvested cells were resuspended in 50 mM Tris HCl (pH 8.0) and 10 mM imidazole and were disrupted by sonication. After centrifugation at 40 000 × g for 30 min, the supernatant was purified using Ni-NTA Superflow (QIAGEN) resin. PhoExo I-CHis was eluted with a buffer solution containing 50 mM Tris HCl (pH 8.0) and 200 mM imidazole. PhoExo I-CHis was further purified with a Resource Q column, as described above.

The expression plasmids for the PhoExo I-CHis mutants (D7N, A11F, E61Q, D80N, K135A, E145Q, R172A, D189R and N214L) were prepared with the PrimeSTAR Mutagenesis Basal Kit (TaKaRa) using the pET26b-PhoExo I-CHis plasmid as a template. The protein expression and purification of the mutants were performed using the same method used for the PhoExo I-CHis preparation.

The heterotrimeric PhoExo I proteins (the wild-type―wild-type―K136A and the wild-type―K136A―K136A heterotrimers) were overexpressed using the pRSF Duet-1 vector (Novagen). Gene fragments of PhoExo I-CHis and PhoExo I K136A with a C-terminal acidic tag (PhoExo I K136A-DEQE) were amplified by PCR and cloned into the NdeI/XhoI site and the NcoI/BamH1 site, respectively. The constructed plasmid was transformed into *E. coli* Rosetta (DE3) for protein expression. The heterotrimeric PhoExo I proteins were expressed using the same method used for the native PhoExo I overexpression. The heterotrimeric PhoExo I proteins were purified with Ni-NTA Superflow (QIAGEN) resin as described above and were further purified using a Mono Q 10/10 (GE Healthcare) column. The bound proteins were eluted with a liner gradient of 0–1 M NaCl in 20 mM Tris HCl (pH 8.0). To analyse components of the purified heterotrimers, the samples were mixed with an equal amount of the solution (10% glycerol and 3.5% SDS); they were then incubated at 95°C for 15 min. The denatured proteins were separated through a denaturing 10% polyacrylamide gel in 0.5× TBE and 7 M urea and were visualized by Coomassie staining. The concentrations of protein bands were quantified using ImageJ software (NIH). To analyse the stability, the purified heterotrimers were incubated at 65°C for 30 min and were analysed using a MonoQ 10/10 column.

### 3′-5′ exonuclease activity assay

The 3′-5′ exonuclease activities of the purified PhoExo I-CHis and its mutants were detected using 5′-fluorescein-labelled 25-nt poly-dT (5′-fluorescein-TTTTTTTTTTTTTTTTTTTTTTTTT-3′), 5′-fluorescein-labelled 26-nt poly-dT (5′-fluorescein-TTTTTTTTTTTTTTTTTTTTTTTTTT-3′), 5′-fluorescein-labelled 25-nt poly-dC (5′-fluorescein-CCCCCCCCCCCCCCCCCCCCCCCCC-3′) and 5′-fluorescein-labelled 25-nt poly-dA (5′-fluorescein-AAAAAAAAAAAAAAAAAAAAAAAAA-3′) as substrates. For the time course analysis of the 3′-5′ exonuclease activity, 100 nM of fluorescein-labelled poly-dT (25- or 26-nt) and 33.3 nM of the PhoExo I-CHis trimer were mixed in 20 μl of 20 mM Tris HCl (pH 8.0), 20 mM NaCl and 2 mM MgCl_2_; they were then incubated at 65°C for 0.5, 1, 2, 4 and 8 min. For the sequence preference assay, 33.3 nM of the PhoExo I-CHis trimer was mixed with 100 nM of fluorescein-labelled poly-dA, fluorescein-labelled poly-dC, or fluorescein-labelled poly-dT (25-nt) in 20 μl of 20 mM Tris HCl (pH 8.0), 20 mM NaCl and 2 mM MgCl_2_; it was then incubated at 65°C for 5 and 10 min. For the 3′-5′ exonuclease activity assay of the PhoExo I mutants, 100 nM of fluorescein-labelled poly-dT (25-nt) and 33.3 nM of the trimers were mixed in 20 μl of 20 mM Tris HCl (pH 8.0), 20 mM NaCl and 2 mM MgCl_2_; the mixture was then incubated at 65°C for 10 min (all constructs) or for 5, 10, 15 and 20 min (the K136A, R172A and N214L mutants). For the Mg^2+^ ion-dependent 3′-5′ exonuclease activity assay, 100 nM of fluorescein-labelled poly-dT (25-nt) and 16.7 nM of the PhoExo I-CHis trimer were mixed in 20 μl of 20 mM Tris HCl (pH 8.0), 50 mM NaCl, and 5–200 mM MgCl_2_ and were incubated at 50°C for 10 min. For the 3′-5′ exonuclease activity assays of the heterotrimeric (the wild-type―wild-type―K136A and the wild-type―K136A―K136A heterotrimers) and the monomeric PhoExo I (D189R mutant), 100 nM of fluorescein-labelled poly-dT (25-nt) was mixed with 33.3 nM of the heterotrimers or 100 nM of the D189R mutant in 20 μl of 20 mM Tris HCl (pH 8.0), 20 mM NaCl and 2 mM MgCl_2_; it was then incubated at 65°C for 0.5, 1, 2, 4, 8 and 16 min. After the enzymatic reactions, the reaction solutions were immediately transferred to ice and supplemented with an equal amount of the solution (10 M urea and 0.1 M EDTA) to stop the enzymatic reactions. The solutions were separated through a denaturing 18% polyacrylamide gel in 0.5× TBE and 7 M urea, and the fluorescence was measured using an LAS4000 Mini system (Fujifilm, Tokyo, Japan).

The RNA/DNA hybrid substrate was prepared by mixing 5′-fluorescein-labelled 30-nt RNA (5′-fluorescein-rCGAACUGCCUGGAAUCCUGACGAACUGUAG) with the complementary DNA strand (dCTACAGTTCGTCAGGATTCCAGGCAGTTCG). The cleavage reactions of the 5′-fluorescein-labelled RNA and the RNA/DNA hybrid were performed with 10 nM and 25 nM of the PhoExo I-CHis trimer in solutions containing 25 nM substrate, 25 mM Tris HCl (pH 8.0), 50 mM NaCl, 5 mM MgCl_2_ and 0.1 mg/ml BSA. After incubation for 5 min at 65°C, the samples were mixed with 20 mM EDTA to stop the reactions and were subjected to 15% PAGE in TBE buffer. The products were visualized using an image analyser, Typhoon Trio+ (GE Healthcare).

The 5′-^32^P-labelled 27-nt RNA (5′-^32^P-rAGCUAUGACCAUGAUUAC GAAUUGCUU) was used to detect the 3′-5′ exonuclease activities of PhoExo I-CHis and its N214L mutant. For the time course analysis, 25 nM of RNA and 8 nM of the trimers were mixed in 25 mM Tris HCl (pH 8.0), 50 mM NaCl, 5 mM MgCl_2_ and 0.1 mg/ml BSA and were incubated at 65°C for 2, 4 and 8 min. The reactions were terminated with a double portion of the stop solution (98% formamide and 10 mM EDTA). The samples were separated by 8 M urea-18% PAGE in TBE buffer. The products were visualized using an image analyser, Typhoon Trio+ (GE Healthcare).

To quantitate the activity of the purified PhoExo I-CHis with RNA and DNA substrates, the amounts of digested products by the PhoExo I-CHis were quantified by measuring the fluorescence strength using Qubit® fluorometric quantitation system (Life Technologies). The oligonucleotides with the same sequence, 5′-dCGAACTGCCTGGAATCCTGACGAACTGTAG-3′ and 5′-rCGAACUGCCUGGAAUCCUGACGAACUGUAG-3′, were used as substrates. The assays were performed with a 40-fold excess molar ratio of substrate over enzyme, as indicated in the legends. The residual amounts of DNA or RNA after cleavage reactions were detected at each time point, and the products were quantified by the calculating formula: digested amounts = initial amounts – residual amounts. The amounts of digestion product were plotted versus reaction time, and the reaction rates were obtained as nucleotides/ second/ trimer protein by using the initial linear portion of the reaction curve. The average rates were obtained from three independent experiments. To analyse the nuclease activity for long DNA and RNA, poly-dT (Amersham Pharmacia Biotech, size is not defined) and RNA marker (SIGMA. 0.28–6.6 kb) were used. *E. coli* Exo I was obtained from Takara Bio. These longer nucleotides were indefinite length and were used for the assay at the same weight as the oligonucleotides.

### Electrophoresis mobility shift assay

The probes, 27-nt RNA (rAGCUAUGACCAUGAUUACGAAUUGCUU) and 27-nt DNA (dAGCTATGACCATGATTACGAATTGCTT) were labelled with ^32^P at their 5′ termini. Various amounts of PhoExo I-CHis and its N214L mutant (0, 25, 50, 100, 200, 400, 800 and 1600 nM as a trimer) were mixed with the probes (5 nM) in 20 μl of 25 mM Hepes NaOH (pH 7.5), 50 mM NaCl, 0.1 mM EDTA and 0.1 mg/ml BSA and were incubated for 5 min at 55°C. Glutaraldehyde was added at a final concentration of 0.05%, and the solutions were incubated for 10 min at room temperature. The reaction was quenched by adding 0.1 M Tris HCl (pH 8.0) and 12% Ficoll at a ratio of 1:4 to the reaction solution. The samples were subjected to 7.5% native PAGE in TBE buffer, and the nucleotides were visualized using Typhoon Trio+ (GE Healthcare).

### Oligomeric state analysis by gel filtration chromatography

The purified proteins were loaded onto a Superdex 200 HR 10/30 (GE Healthcare) column and were eluted with buffer containing 10 mM Tris HCl (pH 8.0) and 200 mM NaCl. To estimate the multimerization state of PhoExo I, the following standard proteins were used: thyroglobulin (*M*_r_ = 669 000), ferritin (*M*_r_ = 440 000), conalbumin (*M*_r_ = 75 000), ovalbumin (*M*_r_ = 44 000), chymotrypsinogen A (*M*_r_ = 25 000) and ribonuclease A (*M*_r_ = 13 700).

### Crystallization and data collection of PhoExo I

The native and Se-Met-labelled PhoExo I were dialysed against 20 mM Tris HCl (pH 8.0) and 0.2 M MgCl_2_ and were concentrated to 6 mg/ml for crystallization. All the crystallization experiments were performed at 20°C using the sitting-drop vapour-diffusion method. Crystals of the native PhoExo I were obtained in a reservoir solution containing 0.1 M Bis-Tris (pH 6.7), 36% polyethylene glycol monomethyl ether 550 and 50 mM CaCl_2_. Crystals of Se-Met-labelled PhoExo I were obtained in a reservoir solution containing 0.1 M Tris HCl (pH 8.3), 3.4 M 1,6-hexanediol and 0.2 M MgCl_2_.

X-ray diffraction data sets of the native and Se-Met-labelled PhoExo I crystals were collected at beamlines BL-5A, AR-NE3A and AR-NW12A of the Photon Factory (Tsukuba, Japan). The X-ray diffraction data sets were collected under cryogenic conditions (95 K) in a nitrogen stream. The diffraction data were indexed, integrated and scaled with XDS (25). The crystals of the native PhoExo I belonged to the space group *P*2_1_2_1_2_1_ with two different unit cell parameters (crystals 1 and 2). The crystal of Se-Met-labelled PhoExo I belonged to the space group *H*32. The crystallization and the data collection of the refolded PhoExo I were performed as described (crystal 3) (22). The data collection statistics of PhoExo I are summarized in Table 1.

**Table 1.** Data collection, phasing and refinement statistics of PhoExo I

Crystal name	Native			Se-Met		
	Crystal 1	Crystal 2	Crystal 3	Peak	Edge	Remote
***Data collection***						
Beamline	BL-5A	BL-5A	AR-NE3A		AR-NW12A	
Space group	*P*2_1_2_1_2_1_	*P*2_1_2_1_2_1_	*H*32		*H*32	
Cell dimensions (Å)						
*a*	94.61	81.94	112.07		112.34	
*b*	94.82	88.98	112.07		112.34	
*c*	99.71	93.95	202.28		203.83	
Wavelength (Å)	1.00000	1.00000	1.00000	0.97923	0.97942	0.96418
Resolution (Å)	20-2.15 (2.21-2.15)*	20-2.20 (2.26-2.20)	20-1.52 (1.60-1.52)		20-2.67 (2.74-2.67)	
*R*_merge_ (%)	8.4 (63.4)	6.7 (54.6)	3.3 (53.6)	6.3 (27.9)	6.7 (35.3)	7.0 (45.3)
Mean *I*/σ*I*	13.1 (2.6)	18.3 (2.0)	30.2 (2.1)	34.8 (8.5)	33.8 (6.9)	34.4 (5.5)
Completeness (%)	99.7 (98.6)	99.5 (97.6)	98.6 (91.1)	98.2 (81.0)	98.5 (86.1)	98.8 (89.6)
Multiplicity	6.8 (4.6)	6.8 (3.8)	6.6 (3.1)	20.7 (12.1)	20.8 (12.4)	20.9 (12.5)
***Phasing***						
Phasing power				2.79 (0.78)	2.50 (0.64)	2.03 (0.54)
Number of Se sites					6	
FOM					0.686 (0.359)	
***Refinement***						
*R*/*R*_free_ (%)	18.2/22.4	19.3/23.3	16.0/18.0	20.9/25.7		
No. atoms						
Protein/Mg^2+^ ion/water	5245/3/236	4972/3/97	3568/2/386	3489/2/74		
*B*-factors (Å^2^)						
Protein/Mg^2+^ ion/water	52.3/54.8/44.0	45.3/50.9/41.6	32.5/22.9/41.3	25.9/32.1/38.0		
R.m.s deviations						
Bond lengths (Å)	0.005	0.003	0.007	0.038		
Bond angles (^o^)	0.949	0.891	1.080	2.843		
Ramachandran plot						
Favoured region (%)	97.4	97.5	98.0	88.5		
Allowed region (%)	2.6	2.3	2.0	8.7		
Outer region (%)	0.0	0.2	0.0	2.8		

*Values in parenthesis are for the highest resolution shell.

### Crystallization and data collection of PhoExo I―ssDNA complexes

To obtain the crystals of the PhoExo I―ssDNA complexes, the purified PhoExo I-CHis D80N mutant and 7-nt ssDNAs (5′-AAAAAAA-3′, 5′-CCCCCCC-3′ and 5′-TTTTTTT-3′) were mixed at a molar ratio of 1:1 and concentrated to 200 μM in 10 mM Tris HCl (pH 8.0) and 5 mM MgCl_2_. Crystals of PhoExo I―ssDNA complexes were obtained in a reservoir solution containing 0.1 M MES (pH 5.6–5.7), 12–14% PEG6000 and 1 M LiCl.

For cryoprotection, the crystals were soaked for a few seconds in reservoir solution supplemented with 40% (v/v) glycerol. To obtain the PhoExo I―poly-dT―Mg^2+^ crystals, the crystals of PhoExo I―poly-dT were soaked for a few seconds in reservoir solution supplemented with 40% glycerol and 0.1 M MgCl_2_. The X-ray diffraction data sets were collected under cryogenic conditions (95 K) in a nitrogen stream, and the diffraction data were indexed and integrated with XDS (25) and scaled with SCALA in CCP4 suite (26). The crystals of PhoExo I in complexes with poly-dA, poly-dC, poly-dT and poly-dT―Mg^2+^ belonged to the space group *P*2_1_2_1_2_1_. The data collection statistics of the PhoExo I―ssDNA complexes are summarized in Table 2.

**Table 2.** Data collection and refinement statistics of PhoExo I―ssDNA complexes

Crystal name	Poly-dA complex	Poly-dC complex	Poly-dT complex	Poly-dT―Mg^2+^ complex
***Data collection***				
Beamline	BL-5A	BL-5A	BL-5A	BL-5A
Space group	*P*2_1_2_1_2_1_	*P*2_1_2_1_2_1_	*P*2_1_2_1_2_1_	*P*2_1_2_1_2_1_
Cell dimensions (Å)				
*a*	94.68	81.57	94.74	94.88
*b*	95.10	100.26	94.75	94.89
*c*	105.27	113.18	105.15	106.46
Wavelength (Å)	1.00000	1.00000	1.00000	1.00000
Resolution (Å)	20-2.05 (2.16-2.05)*	20-2.50 (2.64-2.50)	20-2.05 (2.16-2.05)	20-1.95 (2.06-1.95)
*R*_merge_ (%)	5.8 (48.5)	8.1 (95.8)	7.8 (86.7)	8.8 (84.3)
Mean *I*/σ*I*	17.1 (2.8)	15.9 (2.2)	16.2 (2.5)	13.0 (2.4)
Completeness (%)	99.3 (96.4)	99.3 (97.2)	99.9 (100)	99.9 (100)
Multiplicity	6.7 (4.3)	6.8 (6.0)	7.3 (7.4)	7.0 (6.4)
***Refinement***				
*R*/*R*_free_ (%)	19.1/23.3	20.2/24.0	17.9/20.8	18.0/20.4
No. atoms				
Protein/Mg^2+^ ion/DNA/water	5328/-/252/129	5379/-/228/59	5328/-/240/151	5328/2/240/236
*B*-factors (Å^2^)				
Protein/Mg^2+^ ion/DNA/water	69.5/-/102.8/52.7	68.9/-/90.4/53.7	65.5/-/76.1/50.6	57.3/78.8/75.6/48.3
R.m.s deviations				
Bond lengths (Å)	0.008	0.003	0.008	0.008
Bond angles (o)	1.122	0.718	1.167	1.130
Ramachandran plot				
Favoured region (%)	97.4	97.3	98.0	98.5
Allowed region (%)	2.6	2.7	2.0	1.5
Outer region (%)	0	0	0	0

*Values in parenthesis are for the highest resolution shell.

### Structure determination of PhoExo I

The structure of PhoExo I was determined using the multi-wavelength anomalous dispersion (MAD) method. The selenium sites of the crystal were determined using the program ShelxCD (27). The initial phase was calculated with the program sharp (28) using the selenium site coordinates solved by ShelxCD. The phase calculation resulted in an overall figure of merit (FOM) of 0.686 for the resolution range of 20–2.67 Å. The phase was improved using the programs solomon and DM in CCP4 suite (26). The initial model of the Se-Met-labelled PhoExo I was built using the program ARP/wARP (29). The model was manually rebuilt and refined using the programs COOT and Phenix.refine, respectively (30,31). The initial models of the native PhoExo I structures were determined with the program MOLREP (32) using the coordinates of the Se-Met-labelled PhoExo I structure. The structures of native PhoExo I were refined and rebuilt using the programs COOT and Phenix.refine, respectively (30,31). The geometries of the final structures were evaluated using the program Molprobity (33). The refinement statistics of the PhoExo I structures are summarized in Table 1.

### Structure determination of PhoExo I―ssDNA complexes

The structures of the PhoExo I―ssDNA complexes were determined using the molecular replacement method with the program MOLREP (32) using the coordinates of the native PhoExo I crystal described above. The structure of the PhoExo I―ssDNA complexes were refined and rebuilt using the programs COOT and Phenix.refine, respectively (30,31). The geometries of the final structures were evaluated using the program Molprobity (33). The refinement statistics of the PhoExo I―ssDNA complex structures are summarized in Table 2.

### Computational analysis

The structures of PhoExo I were analysed using a set of computer programs: Dali for the search for similar structures from the database (34); Dalilite for the superposition of molecules (35); PISA for the analysis of protein interfaces, surfaces and assemblies (36); ESpript for the preparation of alignment figures (37); ConSurf for the mapping of the sequence conservation to the protein surface (38); APBS for the calculation of macromolecular electrostatics (39) and Pymol (http://www.pymol.org.) for the depiction of structures.

## RESULTS

### Determination of PhoExo I structure

**Figure 1.**
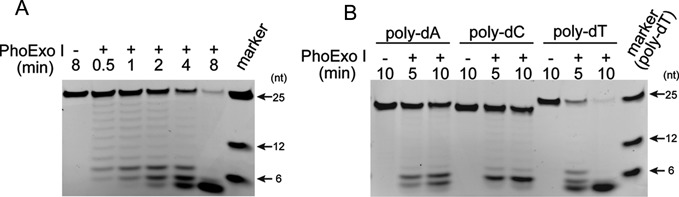
Characterization of PhoExo I. (**A**) Time course analysis of the 3′-5′ exonuclease activity of PhoExo I. The 5′-fluorescein-labelled poly-dT (25-nt, 100 nM) was incubated with PhoExo I (33.3 nM as a trimer) for the indicated times. The products were separated through a denaturing 18% polyacrylamide gel. The 5′-fluorescein-labelled poly-dTs (6-nt, 12-nt and 25-nt) were loaded on the gel to provide markers. (**B**) Base preference of PhoExo I. The 5′-fluorescein-labelled poly-dA, poly-dC and poly-dT (25-nt, 100 nM) were incubated with PhoExo I (33.3 nM as a trimer) for the indicated times. The products were separated through a denaturing 18% polyacrylamide gel.

To reveal the structural basis for the substrate recognition and DNA cleavage mechanisms of PfuExo I and its homologs, we used PhoExo I for structural analyses. PhoExo I forms a trimer in solution similar to PfuExo I (22). The 3′-5′ exonuclease assay of PhoExo I showed that PhoExo I cleaved an ssDNA from its 3′-end at every two nucleotides, and the cleavage stopped at the third to fifth nucleotide from the 5′-end (Figure 1A). Similar to PfuExo I, PhoExo I preferred poly-dT as a substrate over poly-dA and poly-dC (Figure 1B). Because the final products accumulated before the complete degradation of the initial substrates, the cleavage mode of PhoExo I is predicted to be processive for the poly-dT, poly-dA and poly-dC substrates.

The structure of PhoExo I was determined using the MAD method with the selenomethionine (Se-Met) derivative crystal. In this study, we determined three substrate-free PhoExo I structures using three crystals with different unit-cell parameters (crystals 1–3) (Table 1). Crystals 1 and 2 contained one PhoExo I trimer in the asymmetric unit. Crystal 3 contained two PhoExo I protomers in the asymmetric unit. In crystal 3, each protomer of PhoExo I formed a trimer with symmetrically related proteins that were generated by the crystallographic 3-fold axis. The protomer structures of the three crystals were nearly identical. The maximal root mean square deviation (r.m.s.d) between the protomers was 1.2 Å for 199 superposed Cα atoms. Because the structures of PhoExo I in crystals 1 and 2 were partially disordered, we mainly used the structure of PhoExo I in crystal 3 for the images, unless otherwise stated.

### Overall structure of PhoExo I

**Figure 2.**
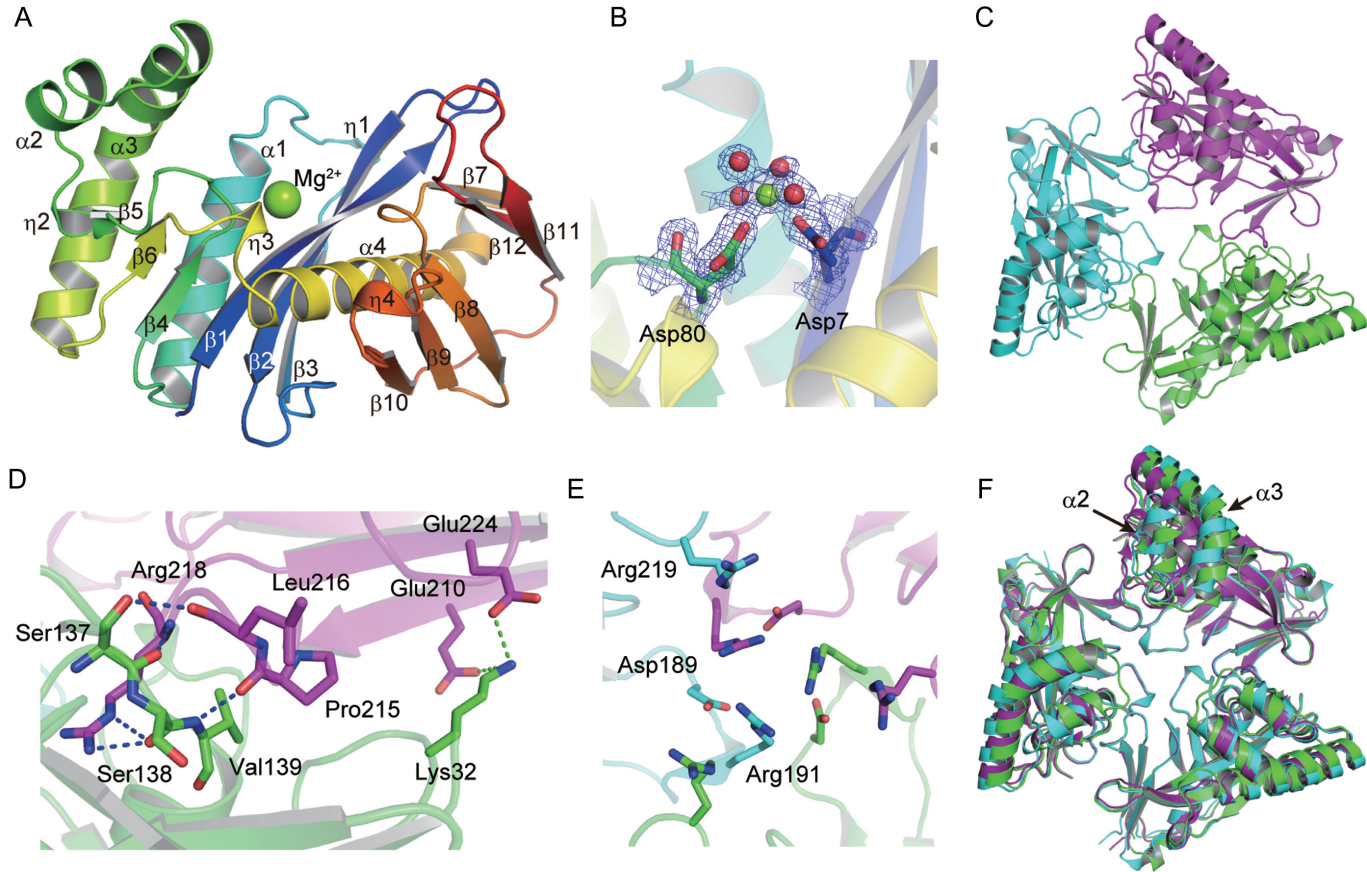
Overall structure of PhoExo I. (**A**) Ribbon diagram of the PhoExo I protomer. The ribbon is coloured blue (in the N-terminus) to red (in the C-terminus). Secondary structure assignments are labelled on the ribbon model. The bound Mg^2+^ ion is shown as a green sphere. (**B**) The composite omit map around the Mg^2+^-binding site is contoured at 1.5 σ (blue mesh). Two acidic residues (Asp7 and Asp80) and four water molecules around the Mg^2+^ ion are shown as stick and sphere models, respectively. The composite omit map was generated using the CCP4 suite (26). (**C**) Trimeric structure of PhoExo I. Three protomers are coloured green, magenta and cyan. (**D**) Interface of the PhoExo I trimer. The PhoExo I protomers are coloured green and magenta. Hydrogen bonds and ion pairs are shown as blue and green dotted lines, respectively. (**E**) Arginine cluster at the 3-fold axis region of the PhoExo I trimer. The PhoExo I protomers are coloured green, magenta and cyan. (**F**) Superposition of the PhoExo I trimers. The trimeric structures in crystals 1, 2 and 3 are coloured cyan, purple and green, respectively.

The structure of PhoExo I comprises 12 β strands, 4 α helices and 4 3_10_ (η) helices (Figure 2A). The longest α helix (α4) is surrounded by a nine-stranded (β1, β2, β3, β4, β5, β6, β7, β11 and β12) mixed β sheet (a large β sheet) and a three-stranded (β8, β9 and β10) antiparallel β sheet (a small β sheet). The other side of the large β sheet faces three α helices (α1, α2 and α3). The α3 helix is bent by 60^o^ in its N-terminal half, causing it to be oriented antiparallel to the α2 helix. In the structures of PhoExo I, each PhoExo I protomer binds one Mg^2+^ ion among the β1 strand of the large β sheet, the α1 helix, the β4–β5 loop and the longest α4 helix. The Mg^2+^ ion is coordinated by Asp7, Asp80 and four water molecules in an octahedral manner (Figure 2B). Because PhoExo I requires Mg^2+^ ions for its 3′-5′ exonuclease activity (22), PhoExo I is predicted to catalyse the hydrolysis of an ssDNA around this magnesium-binding site. PhoExo I forms a hexagram-like trimeric structure using the η3 helix, the N-terminus of the α4 helix, the η4 helix and the β11–β12 loop (Figure 2C). The contact surface area between the two PhoExo I protomers is ∼840 Å^2^ per protomer, which corresponds to 7.5% of the total surface area of PhoExo I. Although the interface area of each protomer is relatively small, the trimerization interfaces are tightly connected by hydrogen bonds and ion pairs (Figure 2D). In the interface region, Pro215, Leu216 and Arg218 of one protomer form five hydrogen bonds with Ser137, Ser138 and Val139 of the other protomer. In addition, Glu210 and Glu224 of one protomer form two ion pairs with Lys32 of the other protomer. It is noteworthy that two arginine residues in each protomer, Arg191 and Arg219, are clustered at the centre of the PhoExo I trimer, and the positive charges of these residues are neutralized by the negative charge of Asp189, which prevents electrostatic repulsion at the 3-fold axis region of the trimer (Figure 2E). Asp189 is predicted to be important for stabilizing the trimeric structure of PhoExo I. In this study, we determined three substrate-free structures of the PhoExo I trimers. Although the protomer structures of each trimer are nearly identical, the trimeric structures are not identical (Figure 2F and Supplementary Figure S2). When the trimeric structures of the three PhoExo I crystals were superposed, the N-terminal half of PhoExo I, especially the α2 and α3 helices, exhibited a structural difference, although the C-terminal half of PhoExo I, which is mainly used for trimerization, was nearly identical between the crystals. In addition, the α2 and α3 helices were partially disordered in the PhoExo I structures in crystals 1 and 2. These data suggest that the trimeric structure of PhoExo I is relatively flexible at its N-terminal half in solution.

### Structural comparison of PhoExo I with other nucleases

**Figure 3.**
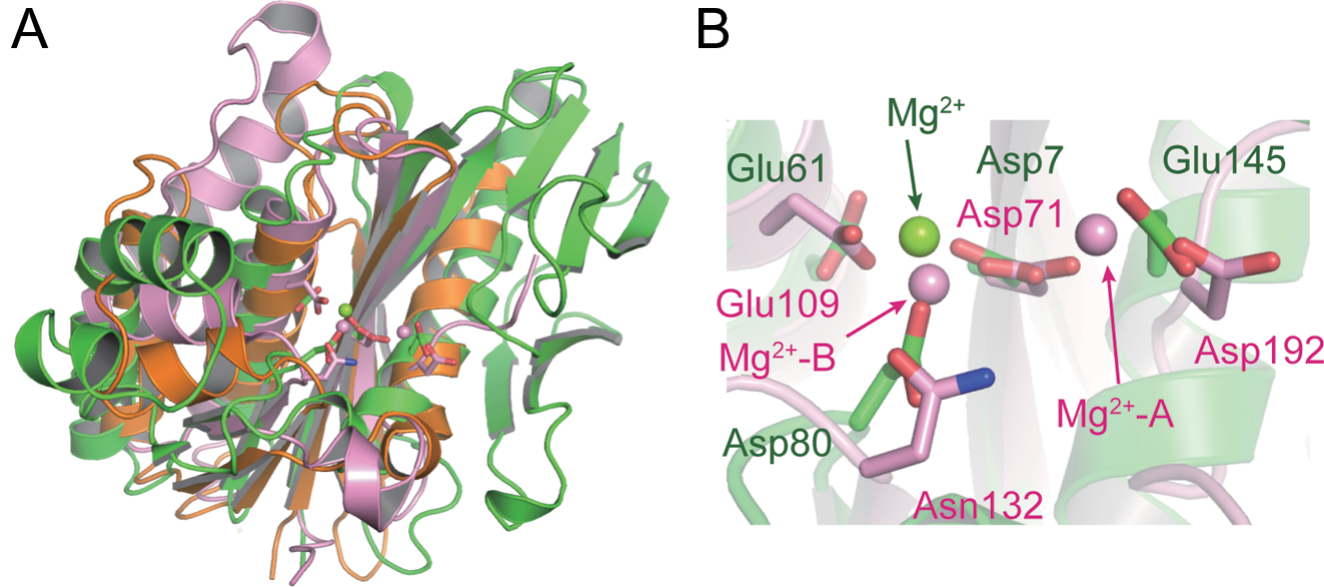
Structural comparison of PhoExo I with RNase H superfamily proteins. (**A**) Superposition of the structures of PhoExo I (green), *S. tokodaii* RNase HI (orange) and *B. halodurans* RNase HI (pink). The Mg^2+^ ions are shown as spheres. Mg^2+^-binding residues are shown as stick models. (**B**) Close-up view of the Mg^2+^-binding sites of PhoExo I and *B. halodurans* RNase HI. Residues from PhoExo I and *B. halodurans* RNase HI are labelled green and pink, respectively.

Because PhoExo I shows no amino acid sequence similarity to any other protein of known function, it was difficult to predict the active site residues and the catalytic mechanism of PhoExo I by performing an amino acid sequence comparison. However, a database search using the Dali server (34) revealed that PhoExo I showed low structural similarity to ribonuclease H (RNase H) family proteins at its centre region (Figure 3A). RNase H is a sequence-nonspecific endonuclease that catalyses the hydrolysis of the RNA strand of RNA/DNA hybrids in the presence of divalent cations. The most similar structure was that of RNase HI from *Sulfolobus tokodaii* (Protein Data Bank (PDB) code: 3ALY, *Z*-score = 8.2, r.m.s.d. = 3.6 Å, sequence identity = 15%) (40). In addition, the structure of RNase HI from *Bacillus halodurans* in complex with an RNA/DNA hybrid (PDB code: 1ZBI, *Z*-score = 4.7, r.m.s.d. = 3.8 Å, sequence identity = 13%) (41) also showed similarity to that of PhoExo I. In the PhoExo I structure, the RNase H fold is sandwiched between the α2 and α3 helices on one side and the C-terminal half of the large β sheet and the small β sheet on the other side. Meanwhile, PhoExo I showed no structural similarity to any exonucleases.

RNase H possesses conserved DEDE (RNase HIII) (42) or DEDD (other RNase Hs) (41,43,44) -type catalytic motifs to hydrolyse the RNA strand. When the structure of PhoExo I was superposed with that of *B. halodurans* RNase HI, the Mg^2+^-binding site of PhoExo I was well superimposed with the active site of *B. halodurans* RNase HI (Figure 3B). Around the Mg^2+^-binding site of PhoExo I, four catalytic residues of *B. halodurans* RNase HI (Asp71; Glu109; Asp 132, which is mutated to Asn in the complex structure, and Asp192) were superposed with Asp7, Glu61, Asp80 and Glu145 of PhoExo I, respectively. PhoExo I is predicted to utilize a DEDE (RNase HIII)-type catalytic motif for the hydrolysis of ssDNA. These acidic residues are highly conserved among the homologs of PhoExo I (Supplementary Figure S1). The Mg^2+^ ion that is coordinated by Asp7 and Asp80 of PhoExo I is located near the B metal ion site of *B. halodurans* RNase HI (Figure 3B). In the *B. halodurans* RNase HI structure, the B metal ion is positioned to stabilize the pentacovalent transition state during the hydrolysis reaction and the 3′-OH of product (41,45). The Mg^2+^ ion bound to the PhoExo I structure would play the same role as the B metal ion of *B. halodurans* RNase HI. In contrast, the binding of a Mg^2+^ ion near the A site was not observed in the PhoExo I structures. The A metal ion is utilized to activate an attacking water molecule (41,45). The second Mg^2+^ ion would be recruited when an ssDNA is bound to the active site of PhoExo I. Similar to the structures of PhoExo I, some structures of RNase H family proteins have also been determined as a single metal ion-binding mode (46,47).

### PhoExo I―ssDNA complexes

To examine the mechanisms of ssDNA recognition and cleavage by PhoExo I, we cocrystallized PhoExo I with poly-dA, poly-dC and poly-dT. To prevent the degradation of ssDNA during the cocrystallization, we used the D80N mutant of PhoExo I; the corresponding mutation was previously utilized in a cocrystallization study of the *B. halodurans* RNase HI―DNA/RNA hybrid complex (41). In this study, we determined the crystal structures of PhoExo I in complexes with poly-dA, poly-dC and poly-dT at 2.05 Å, 2.50 Å and 2.05 Å, respectively. In addition, we determined the crystal structure of the PhoExo I―poly-dT―Mg^2+^ complex at 1.95 Å using the soaking method. Each crystal contained one PhoExo I trimer and three oligonucleotides in the asymmetric unit.

**Figure 4.**
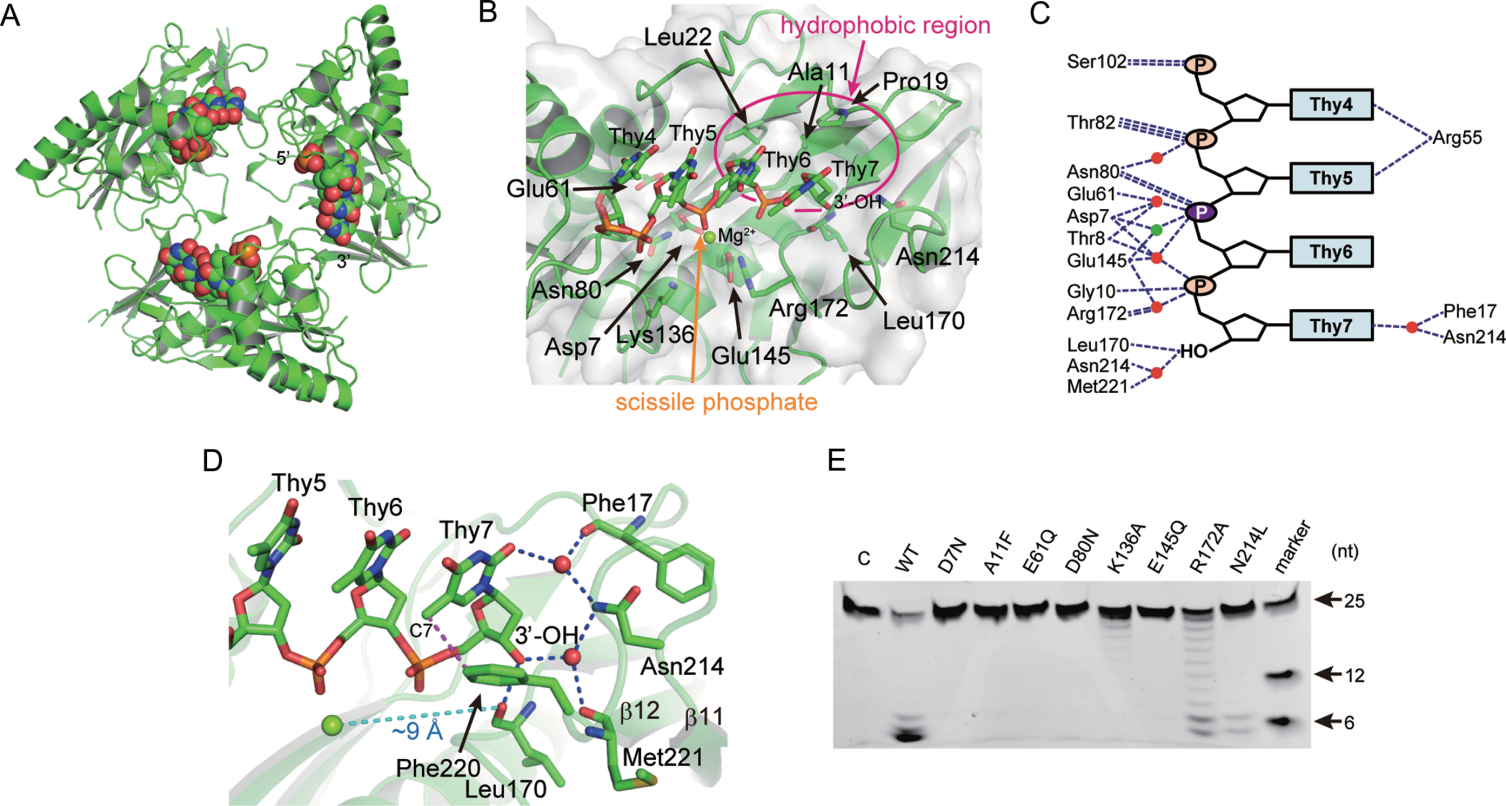
Active site of PhoExo I. (**A**) Trimeric structure of the PhoExo I―poly-dT―Mg^2+^ complex. The structures of PhoExo I and the poly-dT are shown as ribbon and sphere models, respectively. (**B**) Active site pocket of PhoExo I. The hydrophobic region and the scissile phosphate are indicated by pink and orange arrows, respectively. The Mg^2+^ ion is shown as a green sphere. (**C**) Intermolecular hydrogen bonds between PhoExo I and poly-dT in the PhoExo I―poly-dT―Mg^2+^ structure. Hydrogen bonds are shown as blue dotted lines. The Mg^2+^ ion and water molecules are shown as green and red circles, respectively. The scissile phosphate is coloured purple. (**D**) The 3′-OH recognition site of PhoExo I. The Mg^2+^ ion and water molecules are shown as green and red spheres, respectively. Hydrogen bonds are shown as blue dotted lines. A van der Waals contact is shown as a pink dotted line. The distance between the Mg^2+^ ion and the carbonyl oxygen of Leu170 is indicated by a cyan dotted line. (**E**) Mutation analysis. PhoExo I and its mutants (33.3 nM as a trimer) were incubated with the 5′-fluorescein-labelled poly dT (25-nt, 100 nM) for 10 min at 65°C. The products were separated through a denaturing 18% polyacrylamide gel. The 5′-fluorescein-labelled poly-dTs (6-nt, 12-nt and 25-nt) were loaded on the gel to provide markers.

The structure of the PhoExo I―poly-dT―Mg^2+^ complex is shown in Figure 4A and Supplementary Figure S3A. Each PhoExo I protomer binds one poly-dT chain near the Mg^2+^ binding site of PhoExo I. Although we utilized 7 nt of poly-dT for cocrystallization, we only observed electron density at its 3′-terminal 4 nt (Supplementary Figure S3B). These 4 nt of poly-dT are accommodated in a highly conserved Mg^2+^-binding pocket of PhoExo I (Figure 4B and Supplementary Figures S3C and S4). This pocket (the active site pocket) is composed of a hydrophobic region consisting of Ala11, Pro19 and Leu22, the catalytic residues (Asp7, Glu61, Asn80 and Glu145) and the two positively charged residues (Lys136 and Arg172). The deoxyribose groups of the bound poly-dT interact with the hydrophobic region of the pocket. The phosphate groups of the poly-dT are recognized by the catalytic residues and the two positively charged residues. In the PhoExo I―poly-dT―Mg^2+^ complex, one Mg^2+^ ion was observed among Asp7, Glu145 and the phosphate group of Thy6. This position corresponds to the A metal ion site of the *B. halodurans* RNase HI (Figure 3B) (41,45). In contrast, no electron density was observed around the B metal ion site where we observed the Mg^2+^ ions in the substrate-free structures (Supplementary Figure S3B). The D80N mutation would reduce the Mg^2+^ ion-binding affinity at the B metal ion site. The phosphate groups of the poly-dT in the PhoExo I―poly-dT―Mg^2+^ complex are stabilized by eight direct hydrogen bonds and by six indirect (meditated by the Mg^2+^ ion or water molecules) hydrogen bonds (Figure 4C). In the 3′-terminal region of the poly-dT, the 3′-OH is recognized by a hydrogen bond with the main chain carbonyl group of Leu170 and the water-meditated hydrogen bonds with Asn214 and Met221 (Figure 4C and D). Leu170 and Asn214 are highly conserved residues among the homologs (Supplementary Figure S1). Because there is insufficient space to accommodate the 3′-phosphate end of an ssDNA around Leu170 and Asn214, PhoExo I and its homologs could not utilize an ssDNA with a 3′-phosphate end as a substrate (19). Although most exonucleases cleave ssDNA at every nucleotide, PhoExo I cleaves ssDNA at every two nucleotides from the 3′-OH end. This unique feature is explained by the positions of the catalytic residues and the 3′-OH recognition site. In the PhoExo I―poly-dT―Mg^2+^ complex structure, the approximate distance between the bound Mg^2+^ ion at the active site and the main chain carbonyl oxygen of Leu170 is 9 Å. This distance is consistent with the location of the second phosphate group from the 3′-OH end at the active site pocket (Figure 4D). The phosphate groups of Thy4, Thy5 and Thy7 are recognized by direct hydrogen bonds with Ser102, Thr82 and Gly10, respectively (Figure 4C). The phosphate groups of Thy5 and Thy7 are also recognized by one and two water-mediated hydrogen bonds, respectively. Additionally, the phosphate group of Thy6, the scissile phosphate of the poly-dT substrate, forms two direct hydrogen bonds with Asn80 and three indirect hydrogen bonds with Asp7, Thr8, Glu61 and Glu145. Asp7, Glu61, Asp80 and Glu145 of PhoExo I are the DEDE-type catalytic residues of RNase HIII (42), and Thr8 is a highly conserved residue among the PhoExo I homologs (Supplementary Figure S1).

Although the overall structures of the PhoExo I―ssDNA complexes were nearly identical (Supplementary Figure S3D), PhoExo I formed some base-specific interactions with ssDNAs. For example, the C7 methyl group of Thy7 in the PhoExo I―poly-dT and the PhoExo I―poly-dT―Mg^2+^ complexes formed van der Waals interactions with Pho220 (Figure 4D). The electron density maps and the *B*-factors of the corresponding regions indicated that the C7 methyl group of Thy7 was well ordered in the complex structures (Supplementary Figure S3B). In addition, the base groups of the poly-dC and poly-dT formed direct hydrogen bonds with Arg55 and Arg104, and they formed water-mediated hydrogen bonds with Phe17 and Asn214 (Figure 4C and Supplementary Figure S3E, F, G). These interactions may support the substrate recognition of PhoExo I; PhoExo I prefers poly-dT because PhoExo I forms the largest number of base-specific interactions with poly-dT compared to poly-dA and poly-dC. Meanwhile, tracts of adenines are known to exhibit strong base stacking tendencies and increase rigidity of ssDNA (48). Because the active sites of the PhoExo I trimer are covered by the flexible α2 and α3 helices (Figure 2F), it is hard to recruit structurally rigid or partially structured ssDNA to the active sites. PhoExo I may disfavour structurally rigid poly-dA as a substrate.

### Mutation analysis

To investigate the importance of the residues in the active site pocket, we created D7N, A11F, E61Q, D80N, K136A, E145Q, R172A and N214L mutants (Supplementary Figure S5) and analysed their 3′-5′ exonuclease activities. A structural comparison of PhoExo I and the *B. halodurans* RNase HI indicated that Asp7, Glu61, Asp80 and Glu145 are the catalytic residues of PhoExo I. The 3′-5′ exonuclease activity of PhoExo I was completely abolished by the D7N, E61Q, D80N and E145Q mutations (Figure 4E). These residues are predicted to catalyse the hydrolysis of the phosphodiester bond by a two-metal-ion catalytic mechanism similar to the RNase H superfamily (41,45). Lys136 and Arg172, which are located near the B and A metal ion sites, respectively, are conserved positively charged residues in the active site pocket (Figure 4B and Supplementary Figure S1). Although Lys136 formed no direct hydrogen bonds with the phosphate groups of the ssDNAs, except in the PhoExo I―poly-dC complex, the 3′-5′ exonuclease activity of PhoExo I was significantly decreased by the K136A mutation (Figure 4E and Supplementary Figure S6). Arg172 formed a water-mediated hydrogen bond with the phosphate group of Thy7 in the PhoExo I―poly-dT―Mg^2+^ complex. The 3′-5′ exonuclease activity of PhoExo I was moderately decreased by the R172A mutation (Figure 4E and Supplementary Figure S6). These two positively charged residues are predicted to be utilized to retain the negatively charged phosphate groups of an ssDNA at the active site pocket. The structures of the PhoExo I―ssDNA complexes showed that the deoxyribose groups of the substrate DNA contact the hydrophobic region of the active site pocket (Figure 4B). The mutation of Ala11 to a bulky amino acid, Phe, resulted in the loss of the 3′-5′ exonuclease activity (Figure 4E). The A11F mutation is predicted to prevent the binding of an ssDNA to the active site pocket. The 3′-5′ exonuclease activity of PhoExo I was also decreased by the mutation of Asn214, whose side chain recognizes the 3′-OH end of the poly-dT substrate by a water-mediated hydrogen bond (Figure 4D, E and Supplementary Figure S6).

### 3′-5′ exonuclease activities of the heterotrimeric and the monomeric PhoExo I

**Figure 5.**
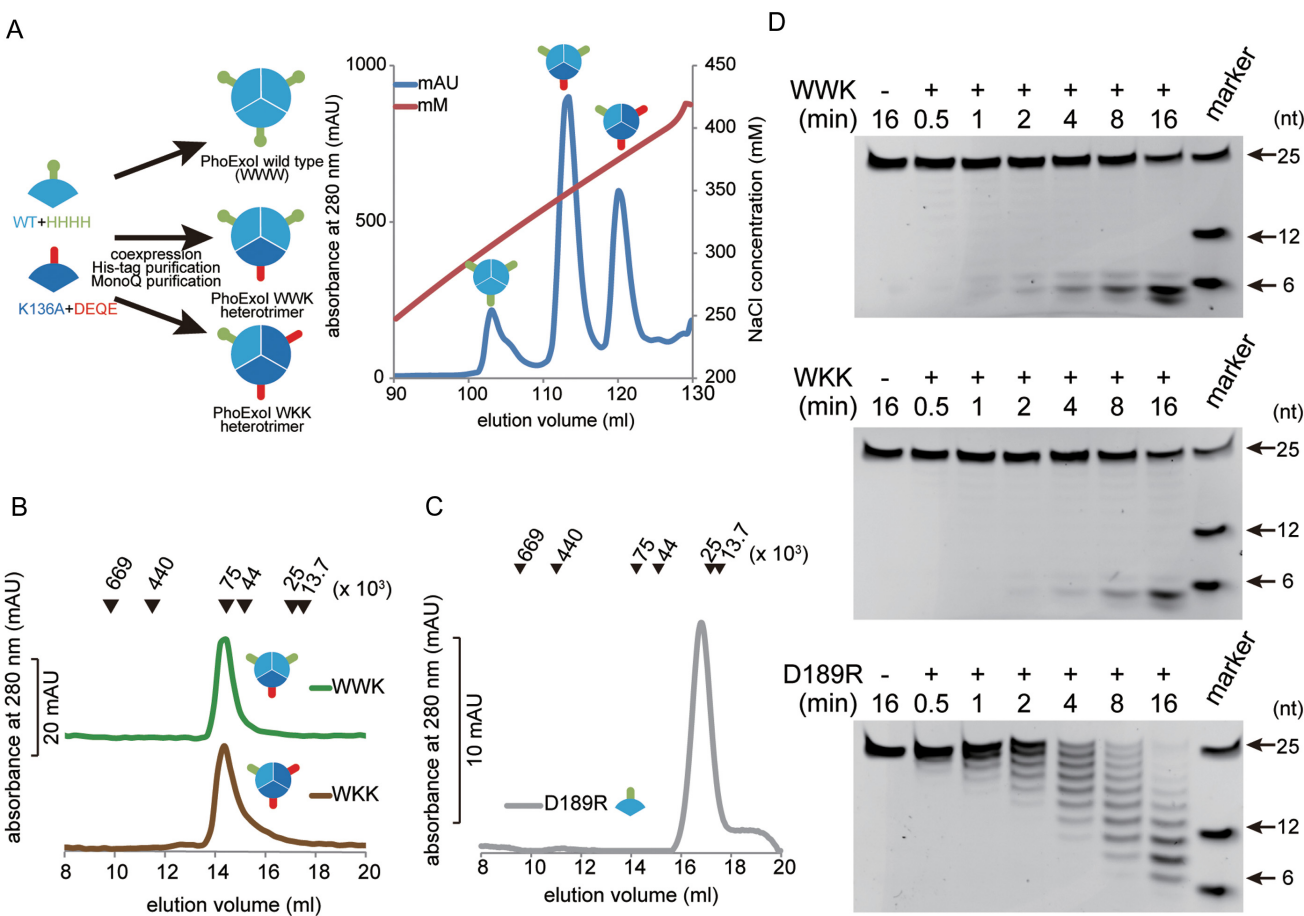
3′-5′ exonuclease activities of the heterotrimeric and the monomeric PhoExo I. (**A**) Preparations of the PhoExo I heterotrimers. The C-terminal His-tagged PhoExo I (cyan) and the C-terminal DEQE-tagged PhoExo I K136A (blue) were coexpressed in *E. coli* and separated using Ni-NTA and anion exchange chromatography (Mono Q) columns (right panel). The heterotrimers were eluted at 340 and 375 mM NaCl. (**B**) Oligomeric state analysis of the PhoExo I heterotrimers by gel filtration chromatography. The peak positions of the marker proteins are indicated by the black triangles at the top of the chromatogram. (**C**) Oligomeric state analysis of the D189R mutant by gel filtration chromatography. (**D**) Time course analysis of the 3′-5′ exonuclease activities of the wild-type―wild-type―K136A heterotrimer (WWK), the wild-type―K136A―K136A heterotrimer (WKK) and the D189R mutant. Each protein (33.3 nM as a trimer) was incubated with the 5′-fluorescein-labelled poly-dT (25-nt, 100 nM) for the indicated times at 65°C. The products were separated through a denaturing 18% polyacrylamide gel. The 5′-fluorescein-labelled poly-dTs (6-nt, 12-nt and 25-nt) were loaded on the gel to provide markers.

Because PhoExo I is a single-strand-specific 3′-5′ exonuclease that functions as a trimer, PhoExo I possesses three identical active sites in its biological unit. To investigate the effect of trimerization on the DNA cleavage activity of PhoExo I, we prepared heterotrimeric and monomeric PhoExo I and analysed their 3′-5′ exonuclease activities. The heterotrimeric PhoExo I proteins were prepared by coexpressing a C-terminal 4xHis-tagged wild-type PhoExo I and a C-terminal acidic (DEQE)-tagged K136A mutant (Figure 5A). The expressed proteins were purified and separated using His-tag purification and anion-exchange chromatography. A gel filtration analysis showed that each separated PhoExo I formed a trimer in solution, similar to the wild-type PhoExo I (Figure 5B). The separated proteins are predicted to be the wild-type―wild-type―K136A (WWK) and the wild-type―K136A―K136A (WKK) heterotrimers of PhoExo I (Supplementary Figure S7A and B). The heterotrimers were not re-equilibrated into other trimers (WWW, WWK, WKK and KKK trimers) after incubation at 65°C for 30 min (Supplementary Figure S7C and D). The monomeric PhoExo I was prepared by creating a point mutation, D189R. The D189R mutation would prevent the trimerization of PhoExo I via the electrostatic repulsion of the Arg residues at the 3-fold axis region (Figure 2E). A gel filtration analysis showed that the D189R mutant existed as a monomer in solution (Figure 5C).

The results of the 3′-5′ exonuclease activity assays of the heterotrimeric and monomeric PhoExo I proteins are shown in Figure 5D. If PhoExo I used a sequential mechanism in which the 3′-OH end of an ssDNA moves cyclically from one active site to the next active site for each round of cleavage, the 3′-5′ exonuclease activities of the WWK and WKK heterotrimers would be significantly decreased because the processive cleavage would halt at the K136A protomer of the heterotrimers. However, the 3′-5′ exonuclease assays showed that the WWK and WKK heterotrimers produced end products (3–5 nt) before the complete degradation of the initial substrate, similar to the wild-type PhoExo I, indicating that the two heterotrimers retained processive DNA cleavage activity. Furthermore, the 3′-5′ exonuclease assay of the D189R mutant showed that the monomerization of PhoExo I resulted in the modification of the cleavage mode from ‘processive’ to ‘distributive’ because the initial substrate was gradually cleaved before the accumulation of the final product. The trimerization of PhoExo I is predicted to be indispensable for the processive cleavage of ssDNA, but only one active site of the trimer is required for the processive cleavage.

Although the WWK and WKK heterotrimers possess two and one wild-type active sites in their heterotrimers, the 3′-5′ exonuclease activities of the heterotrimers were not about two-thirds and one-third of the wild-type homotrimer; the activities of the heterotrimers were reduced more than expected (Figures 1A and 5D). These results indicated that the existence of the K136A protomer reduced the activities of the heterotrimers. Because the K136A mutant retained the 3′-5′ exonuclease activity (Supplementary Figure S6), the K136A protomer does not lose its DNA binding ability. If a substrate ssDNA is trapped by the K136A protomer, the heterotrimer cannot cleave it effectively, until the ssDNA rebinds to a wild-type protomer. This mechanism would reduce the activities of the heterotrimers. A similar mechanism was also observed in the study of the heterotrimeric λ exonuclease activity (49).

### RNase activity of PhoExo I

PfuExo I was originally discovered by screening 3′-5′ exonucleases in *P. furiosus* cells, and its homolog, PhoExo I, has been characterized as a DNase (19,22). However, our crystal structures suggested that PhoExo I may also function in cleaving single-stranded RNA (ssRNA) because the side chain of Asn214, which recognizes the 3′-OH end of ssDNA by a water-mediated hydrogen bond, may interact with a 2′-OH end of an ssRNA to stabilize the enzyme-substrate complex (Figure 4D). Therefore, we analysed the RNA-binding affinity and the RNA cleavage activity of PhoExo I. A 3′-5′ exonuclease assay using an ssRNA showed that PhoExo I efficiently cleaved the ssRNA. However, despite the structural similarity between PhoExo I and RNase H, the RNA was never cleaved when it was hybridized with the complementary DNA (Supplementary Figure S8A). Electrophoresis mobility shift assays (EMSA) using DNA and RNA probes with the exactly same sequences and lengths showed that PhoExo I bound to both DNA and RNA, with an even stronger affinity for RNA (Supplementary Figure S8B). To further investigate the function of Asn214 in substrate recognition, the DNA- and RNA-binding abilities and the RNase activity of the N214L mutant were compared with those of the wild-type PhoExo I. The EMSA and the 3′-5′ exonuclease assay showed that the N214L mutation clearly reduced the DNA- and RNA-binding abilities and the RNA cleavage activity of PhoExo I (Supplementary Figure S8B and C).

To quantitate the activity of PhoExo I for DNA and RNA oligonucleotides, the amounts of the digested products for each reaction time were plotted (Supplementary Figure S9A), and the reaction rates were calculated as 0.210 ± 0.003 nt mol^−1^ s^−1^, 2.50 ± 0.03 nt mol^−1^ s^−1^ for DNA and RNA, respectively. To know the function of PhoExo I under more physiological-like conditions, long nucleotides were used for the nuclease assay. The poly-dT (size was not defined, but was longer than 1000 nt from the denaturing PAGE) was used as the long DNA substrates and nuclease reactions were compared for *E. coli* Exo I (EcoExo I) and PhoExo I. As shown in Supplementary Figure S9B, PhoExo I acted to cleave long DNA strands as EcoExo I did. The reaction rates for long DNA and RNA were compared. The RNA with 0.28–6.6 kb long was used as long RNA substrates. The reaction rates were 1.93 ± 0.12 nt mol^−1^ s^−1^ and 13.4 ± 0.2 nt mol^−1^ s^−1^ for the poly DNA and poly RNA, respectively (Supplementary Figure S9C). Reactions were performed at 85°C for this quantitative analysis to avoid the formation of the secondary structure of RNA. These experiments clearly showed that RNAs were digested faster than DNA, and RNA is probably more preferable substrate for PhoExo I in the *Pyrococcus* cells.

## DISCUSSION

PhoExo I uses the RNase HIII-type active site to hydrolyse the phosphodiester bonds of ssDNA. Although PhoExo I shows structural similarity to RNase H, which cleaves the RNA strand in an RNA/DNA hybrid, the activity of PhoExo I is completely different from that of RNase H due to the PhoExo I-specific regions that surround the RNase H fold. First, PhoExo I could not cleave a dsDNA (19) and an RNA/DNA hybrid, although RNase H utilizes an RNA/DNA hybrid as a substrate (41). This difference can be explained by the location of the active site of PhoExo I. Because RNase H acts as a monomer, the active site and the substrate recognition site of RNase H are highly exposed to solvent. In contrast, the active sites of the PhoExo I trimer are covered by the PhoExo I-specific flexible α2 and α3 helices (Figure 2F). To access the active site of the PhoExo I trimer, a substrate DNA must pass through the 3-fold axis region surrounded by the flexible α2 and α3 helices (the maximal width is ∼25 Å) or through the area between the two protomers (the maximal width is ∼15 Å) (Supplementary Figure S10). Because these spaces are too narrow for double-stranded nucleic acids to access the active site pockets, PhoExo I only utilizes single-stranded nucleic acids, which are structurally more flexible and smaller, as substrates. Second, PhoExo I is an exonuclease that cleaves single-stranded nucleic acids from the 3′-OH end, whereas RNase H is a sequence-nonspecific endonuclease. Because PhoExo I possesses 3′-OH recognition sites (Leu170, Asn214 and Met221) located ∼9 Å away from the active site, PhoExo I cleaves ssDNAs from their 3′-OH end at every two nucleotides. This 3′-OH recognition site is located in the PhoExo I-specific C-terminal region (the C-terminal half of the large β sheet and the small β sheet). Finally, the nuclease activities under high Mg^2+^ ion concentration conditions differ between PhoExo I and RNase H. The activity of RNase H is attenuated by high concentrations of Mg^2+^ ion (50) because the high concentration of Mg^2+^ ion inhibits the product release (45). In the *B. halodurans* RNase HI structure, Glu188 supports the release of the Mg^2+^ ion at the A metal ion site that stabilizes the 5′-phosphate end of the product. Although PhoExo I utilizes an RNase HIII-type active site to hydrolyse phosphodiester bonds, PhoExo I does not possess a residue that corresponds to Glu188 of the *B. halodurans* RNase HI. The release of the Mg^2+^ ion at the A metal ion site is not predicted to be important for the cleavage mechanism of PhoExo I. Actually, the activity of PhoExo I is not inhibited by high concentrations of Mg^2+^ ion (Supplementary Figure S11). Although the RNase H fold is also found in other nucleases, such as retroviral integrase (51), DNA transposase (52), RuvC Holliday junction resolvase (53) and Argonaute in RNA silencing (54–56), PhoExo I shares no structural or functional similarities to these proteins. PhoExo I is a novel RNase H superfamily protein.

**Figure 6.**
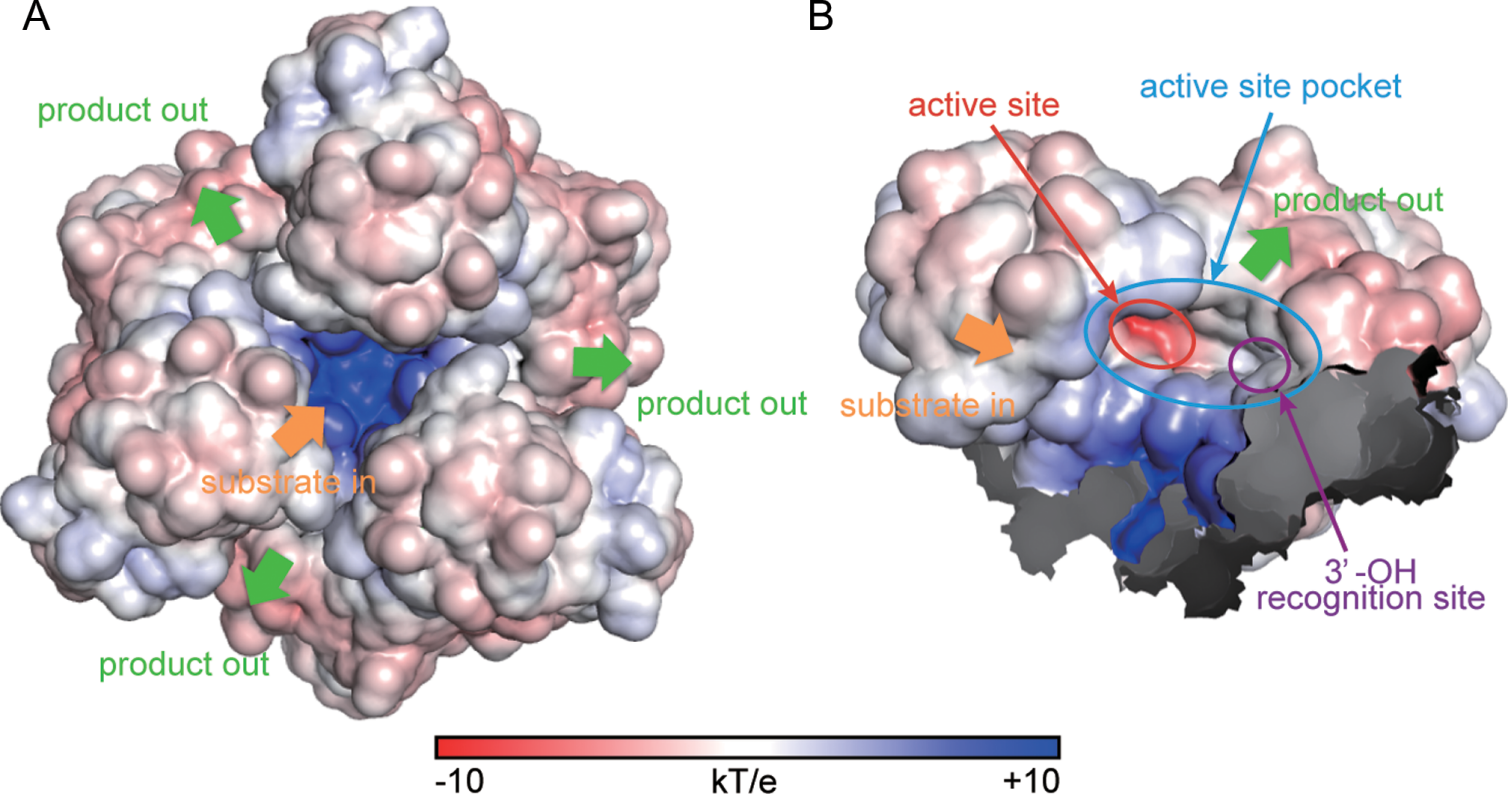
Electrostatic potential of PhoExo I. (**A**) The ±10 kT/e electrostatic potential of the PhoExo I trimer plotted on the solvent-accessible surface. The estimated translocation of an ssDNA is indicated by arrows. (**B**) The ±10 kT/e electrostatic potential of the PhoExo I protomer plotted on the solvent-accessible surface. The active site, the active site pocket and the 3′-OH recognition site are indicated by red, cyan and purple circles, respectively.

**Figure 7.**
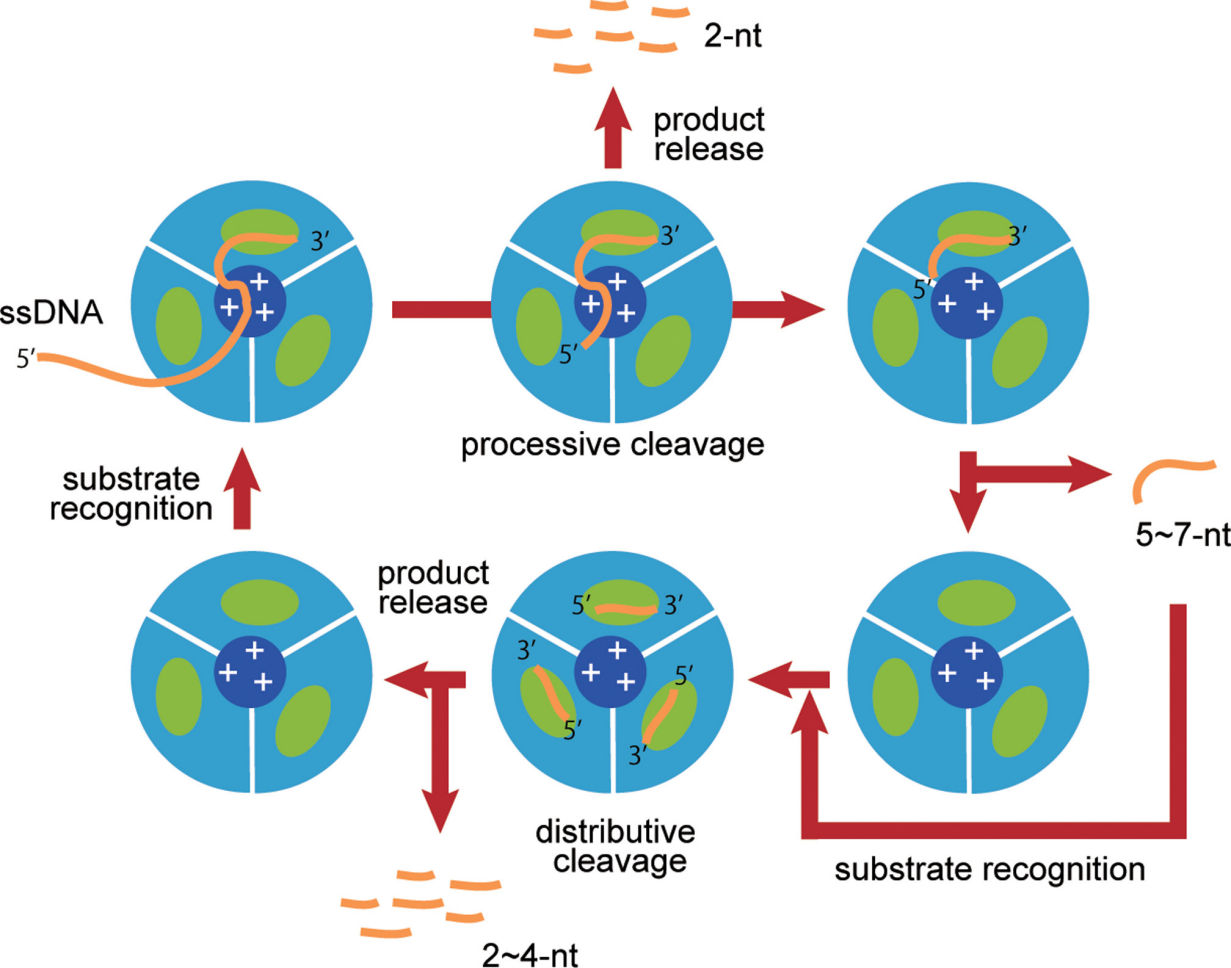
Plausible mechanism of processive cleavage by PhoExo I. Each PhoExo I protomer is shown as a cyan sector. The active site pocket and the positively charged surface of the 3-fold axis region are shown as green and blue circles, respectively. ssDNAs are shown as orange lines.

Because PhoExo I is a trimeric exonuclease, its biological unit contains three independent active site pockets. Although each active site pocket of the protomer binds one ssDNA in the structures of the PhoExo I―ssDNA complexes (Figure 4A and Supplementary Figure S3), the PhoExo I trimer is predicted to bind only one long ssDNA using one of three active sites due to the narrow gateway to the active sites; if each active site binds a long ssDNA at the same time, the 5′-terminal region of each ssDNA strand would be crushed at the 3-fold axis region. The 3′-5′ exonuclease assays of the heterotrimeric and monomeric PhoExo I showed that only one active site of the trimer is required but that trimerization is indispensable for processive ssDNA cleavage (Figure 5D). These data suggest that the arginine cluster at the 3-fold axis region, rather than the three active sites of the trimer, is important for stabilizing the enzyme-substrate complex for processive DNA cleavage. The negatively charged phosphate groups of a long ssDNA substrate would be attracted to the positively charged surface of the arginine cluster at the 3-fold axis region (Figures 2E and 6A, and Supplementary Figure S12A). Once a long ssDNA is captured by the positively charged surface at the 3-fold axis region, four 3′-terminal nucleotides of the ssDNA would be attracted to the active site pocket by the positive charges of Lys136 and Arg172, and the second phosphodiester bond from the 3′-end would be hydrolysed by the RNase HIII-type catalytic residues. The dinucleotide product would be released through the area between the two protomers (Figure 6B and Supplementary Figure S12B). Because Lys136, whose mutant showed significantly decreased activity, is located near the third phosphate group from the 3′-end (Figure 4B), Lys136 can retain the ssDNA after the hydrolysis using its positively charged side chain. The cleaved ssDNA would again be attracted to the same active site pocket by the positive charge of Arg172 for the next round of cleavage. Because the positively charged surface of the 3-fold axis region is located ∼12 Å away from the active site of each protomer (the distance between the ZN nitrogen of Lys136 and the NH2 nitrogen of Arg191 in the PhoExo I―poly-dT―Mg^2+^ complex structure), a short ssDNA would not be captured by both the positively charged surface of the 3-fold axis region and the active site pocket. When an ssDNA becomes sufficiently short to avoid capture by the positively charged surface of the 3-fold axis region after multiple rounds of cleavage, the short ssDNA would be released from the PhoExo I trimer because the stabilization of the enzyme-substrate complex using the positively charged surface in the 3-fold axis region is important for processive cleavage. PhoExo I would cleave the short substrate ssDNA in a distributive manner until the ssDNA becomes sufficiently short to avoid recognition by one active site of the trimer (Figure 7). These plausible cleavage mechanisms, which are based on the structural and biochemical studies, agree well with the results of the 3′-5′ exonuclease assay. When the 25 nt of poly-dT was cleaved by PhoExo I, 5–7 nt of the 5′-labelled products initially accumulated; these 5–7 nt were gradually cleaved to a length of 3 nt (Figure 1A). When the 26 nt of poly-dT were cleaved by PhoExo I, 6 nt of the 5′-labelled product initially accumulated; these 6 nt were gradually cleaved to a length of 4 nt (Supplementary Figure S13). These observations suggest that PhoExo I cleaves ssDNA in a processive manner until the length of the ssDNA becomes 7–9 nt (7 nt of ssDNA is insufficient for the complete processive cleavage, i.e. a part of 7 nt of ssDNAs are released from PhoExo I); the short (5–7 nt) ssDNA is then cleaved in a distributive manner until the length becomes 4 nt or less. The PhoExo I―ssDNA structures determined in this study are predicted to present the distributive cleavage state of PhoExo I. In this state, PhoExo I recognizes ssDNA only at the active site pocket that accommodates the 3′-OH terminal 4 nt of ssDNA, so the 5′-terminal 3 nt are disordered in the complex structures.

Among the exonucleases, λ exonuclease also forms a trimeric structure and cleaves substrate DNA in a processive manner. Although both PhoExo I and λ exonuclease form trimeric structures, the structural bases for their processive DNA cleavage mechanisms are not identical. A structural analysis of λ exonuclease―dsDNA complexes has indicated that the processivity of λ exonuclease is exerted not only at the level of the trimer but also at the level of the monomer (23,49). λ exonuclease uses the positively charged pocket at the end of the active site pocket to attract the 5′-phosphate end of the substrate DNA (23). In contrast, the processivity of PhoExo I is exerted only at the level of the trimer because the positively charged surface of the 3-fold axis region is important for retaining the ssDNA near the active site. The trimer-based processive DNA cleavage mechanism observed in this study is a unique feature of PhoExo I.

## ACCESSION CODE

Coordinates and structural factors are deposited in the PDB under the accession codes 4YOT (native crystal 1), 4YOU (native crystal 2), 4YOR (native crystal 3), 4YOV (poly-dA complex), 4YOW (poly-dC complex), 4YOX (poly-dT complex) and 4YOY (poly-dT―Mg^2+^ complex).

## Supplementary Material

SUPPLEMENTARY DATA
