# Supplementary material for: Structural basis for substrate recognition and processive cleavage mechanisms of the trimeric exonuclease PhoExo I
Source: Nucleic Acids Res. 2015 Jul 2;43(14):7122–36. doi: 10.1093/nar/gkv654 (PMC4538837; doi:10.1093/nar/gkv654)
Supplement: SUPPLEMENTARY DATA [file supp_43_14_7122__index.html]

Structural basis for substrate recognition and processive cleavage mechanisms of the trimeric exonuclease PhoExo I — Structural basis for substrate recognition and processive cleavage mechanisms of the trimeric exonuclease PhoExo I — SUPPLEMENTARY DATA 

# Structural basis for substrate recognition and processive cleavage mechanisms of the trimeric exonuclease PhoExo I

## SUPPLEMENTARY DATA

- SUPPLEMENTARY DATA
