# Supplementary material for: Structural basis for substrate recognition and processive cleavage mechanisms of the trimeric exonuclease PhoExo I
Source: Nucleic Acids Res. 2015 Jul 2;43(14):7122–36. doi: 10.1093/nar/gkv654 (PMC4538837; doi:10.1093/nar/gkv654)
Supplement: SUPPLEMENTARY DATA [file supp_gkv654_nar-00911-h-2015-File011.pdf]

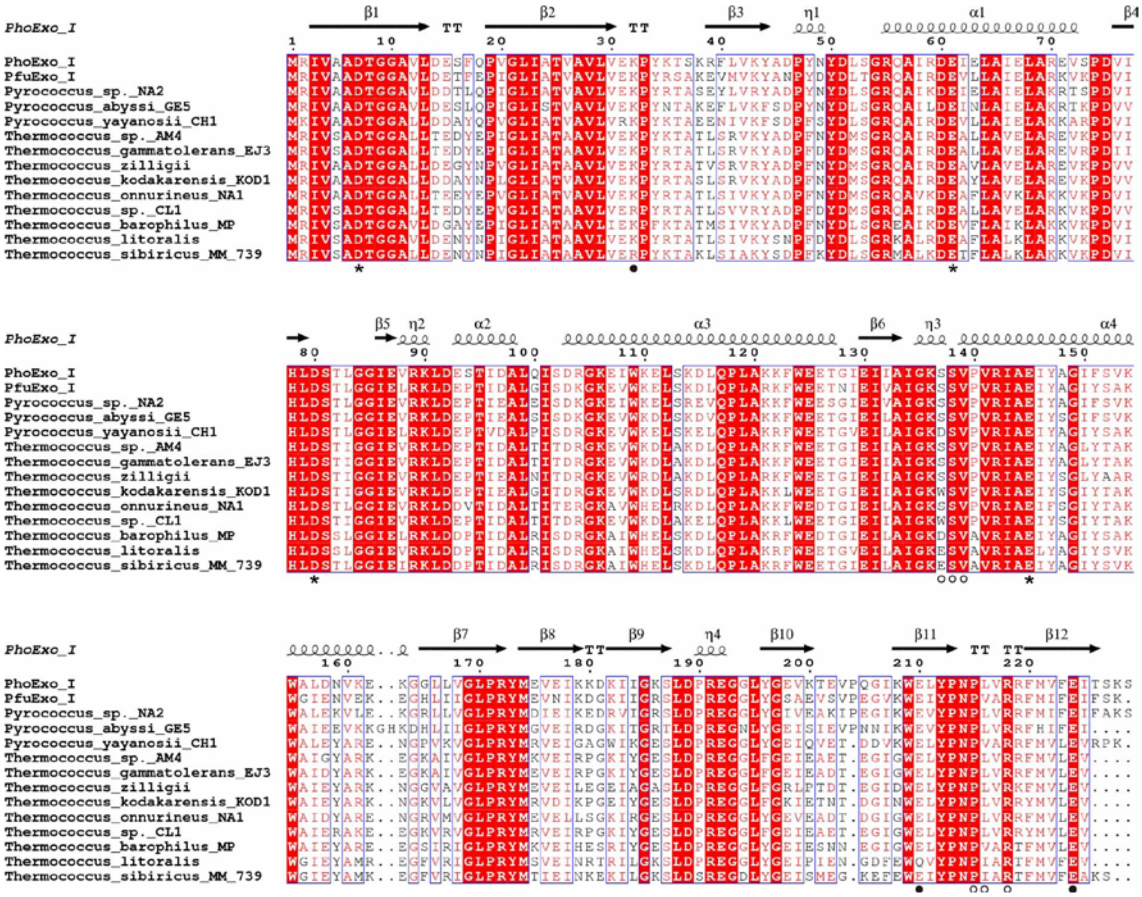

Supplementary Figure 1. Amino acid sequence alignment of PhoExo I, PfuExo I, and their homologs. Invariant residues are highlighted with red boxes, and conserved residues are shown in red text. The secondary structure assignment for PhoExo I is indicated by helices (α and 3_{10}(η) helices), arrows (β strands), and TT (β-turn). The catalytic residues are indicated by asterisks. Residues that form intermolecular hydrogen bonds and ion pairs are indicate by open and filled circles, respectively.

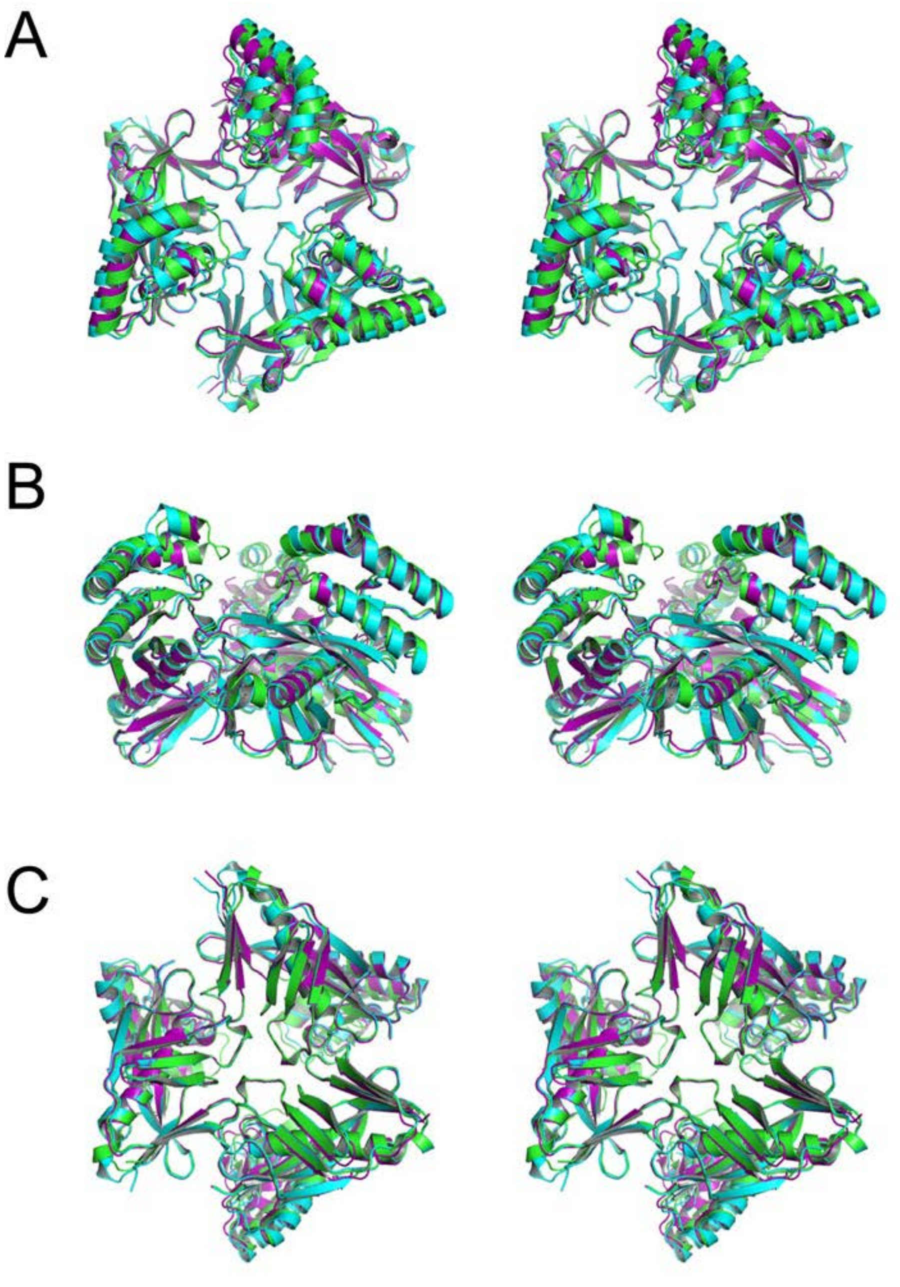

Supplementary Figure 2. Stereo views of the PhoExo I trimers. (A) Wall-eyed stereo image of Figure 2F. (B), (C) Side and back views of (A), respectively.

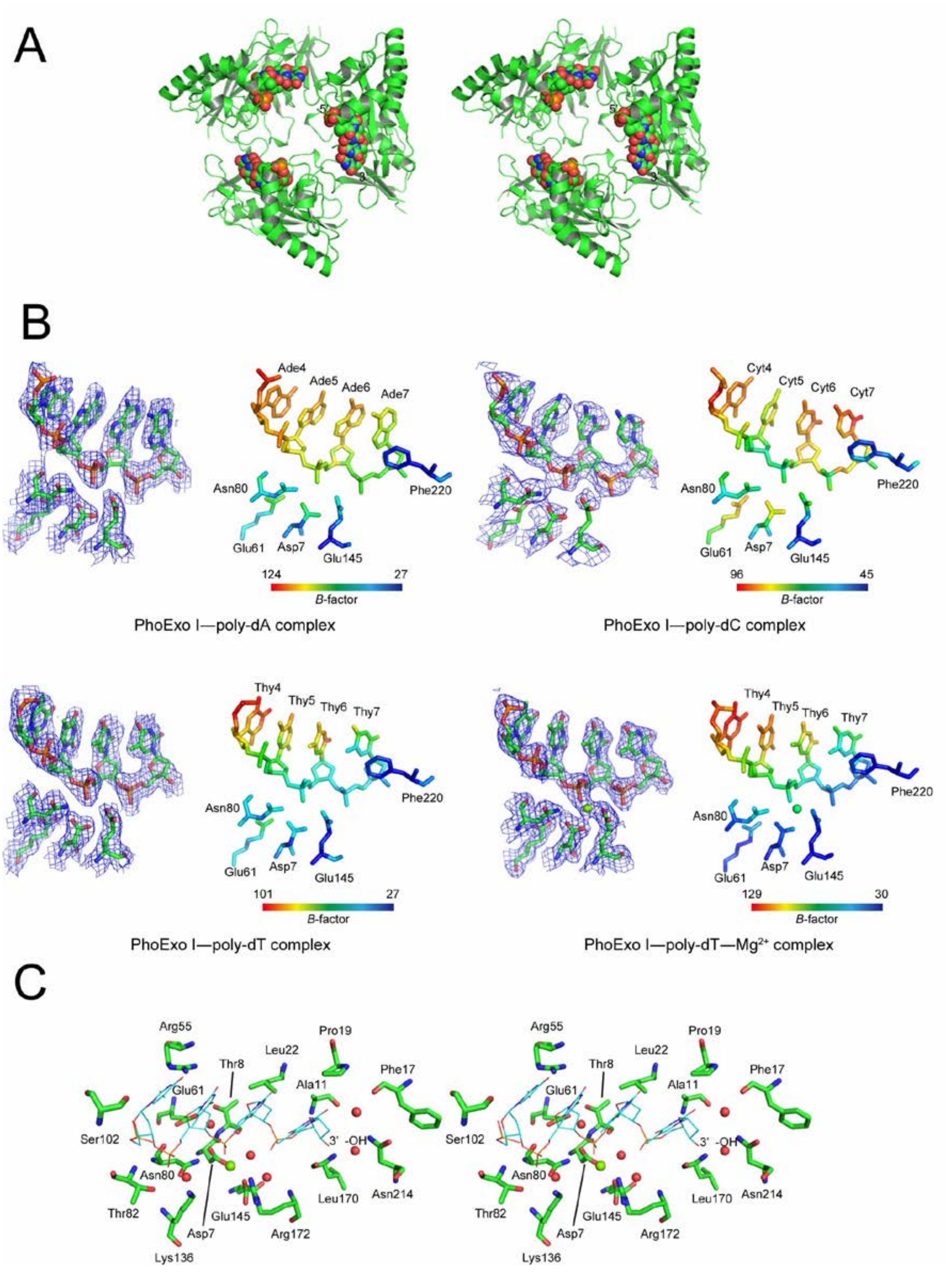
A
5'
3'
5'
3'
B
Ade4
Ade5
Ade6
Ade7
Asn80
Phe220
Glu61
Asp7
Glu145
124
27
B-factor
PhoExo I—poly-dA complex
Cyt4
Cyt5
Cyt6
Cyt7
Asn80
Phe220
Glu61
Asp7
Glu145
96
45
B-factor
PhoExo I—poly-dC complex
Thy4
Thy5
Thy6
Thy7
Asn80
Phe220
Glu61
Asp7
Glu145
101
27
B-factor
PhoExo I—poly-dT complex
Thy4
Thy5
Thy6
Thy7
Asn80
Phe220
Glu61
Asp7
Glu145
129
30
B-factor
PhoExo I—poly-dT—Mg2+ complex
C
Arg55
Pro19
Thr8
Leu22
Phe17
Glu61
Ala11
Ser102
3' -OH
Asn80
Thr82
Leu170
Asn214
Glu145
Asp7
Arg172
Lys136
Arg55
Pro19
Thr8
Leu22
Phe17
Glu61
Ala11
Ser102
3' -OH
Asn80
Thr82
Leu170
Asn214
Glu145
Asp7
Arg172
Lys136

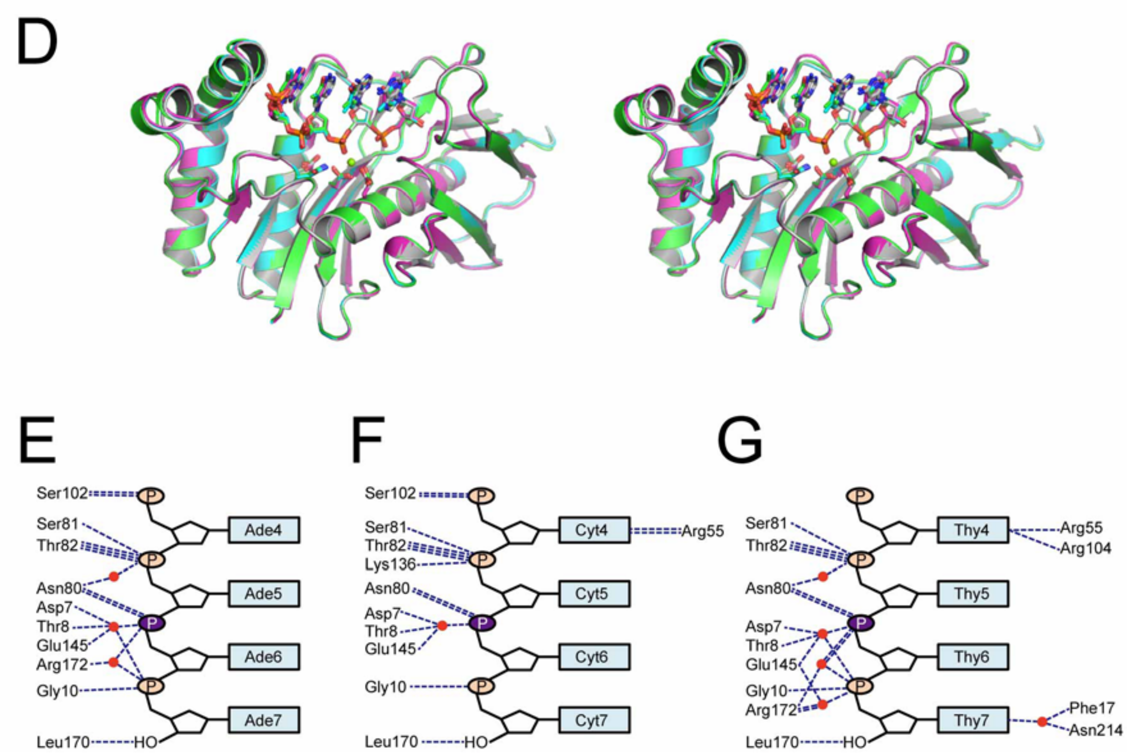

Supplementary Figure 3. Structures of PhoExo I—ssDNA complexes. (A) Wall-eyed stereo image of Fig. 4A. (B) The composite omit maps (blue mesh, 1.0 σ) and the *B*-factors of the PhoExo I—ssDNA complexes. The catalytic residues (Asp7, Glu61, Asn80, and Glu145), Phe220, and ssDNAs are shown as stick models. The Mg^{2+} ion is shown as a sphere. (C) Wall-eyed stereo image of the DNA interacting residues in the PhoExo I—poly-dT—Mg^{2+} complex. The structures of PhoExo I and the poly-dT are shown as stick and line models, respectively. The Mg^{2+} ion and water molecules are shown as green and red spheres, respectively. (D) Superposition of the PhoExo I—poly-dT—Mg^{2+} complex (green), the PhoExo I—poly-dA complex (gray), the PhoExo I—poly-dC complex (magenta), and the PhoExo I—poly-dT complex (cyan) structures (wall-eyed stereo image). (E), (F), (G) Intermolecular hydrogen bonds between PhoExo I and ssDNAs in the PhoExo I—poly-dA, PhoExo I—poly-dC, and PhoExo I—poly-dT complexes. Hydrogen bonds are shown as blue dotted lines. Water molecules are shown as red circles. The scissile phosphate is colored purple.

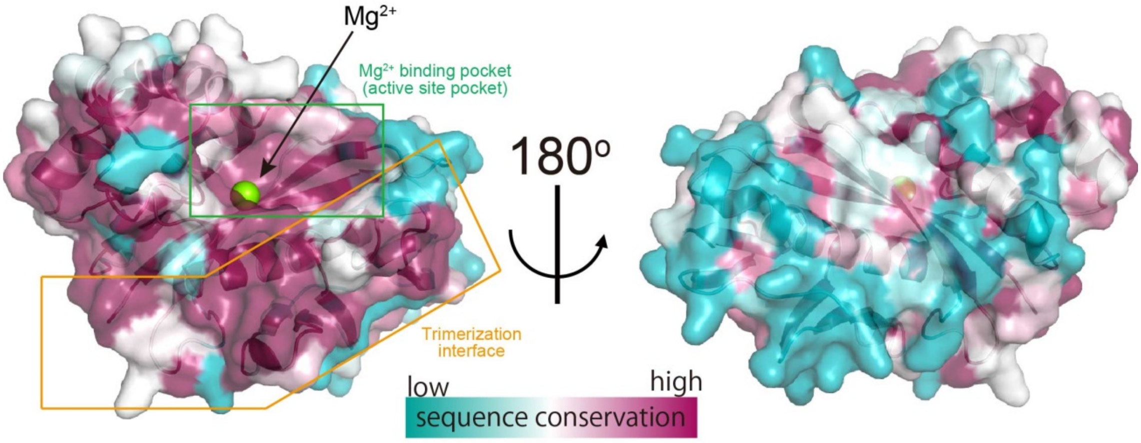

Supplementary Figure 4. Sequence conservation of the PhoExo I surface. The sequence conservation of Supplementary Figure 1 is superimposed on the molecular surface of the PhoExo I protomer.

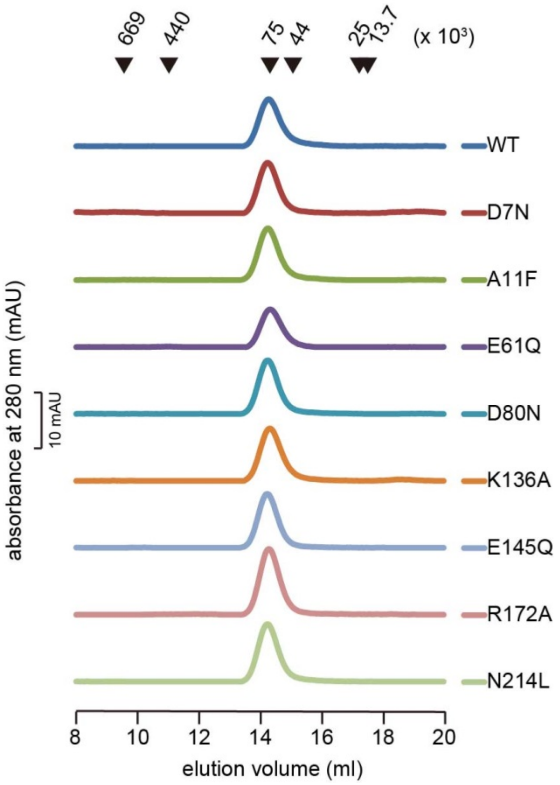

Supplementary Figure 5. Gel filtration analysis of PhoExo I and its mutants. The peak positions of the marker proteins are indicated by the black triangles at the top of the chromatogram. Each mutant formed a trimer in solution.

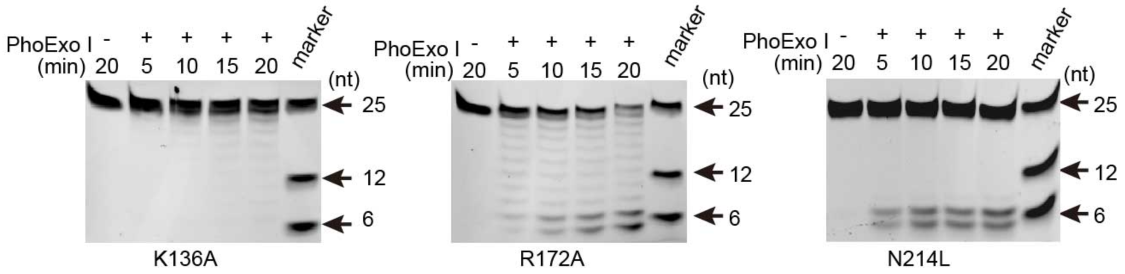

Supplementary Figure 6. Time course analysis of the 3’-5’ exonuclease activity of the K136A, R172A, and N214L mutants. Each mutant (33.3 nM as a trimer) was incubated with the 5’-fluorescein-labeled poly dT (25-nt, 100 nM) for 5, 10, 15, and 20 min at 65°C. The products were separated through a denaturing 18% polyacrylamide gel. The 5’-fluorescein-labeled poly-dTs (6-nt, 12-nt, and 25-nt) were loaded on the gel to provide markers.

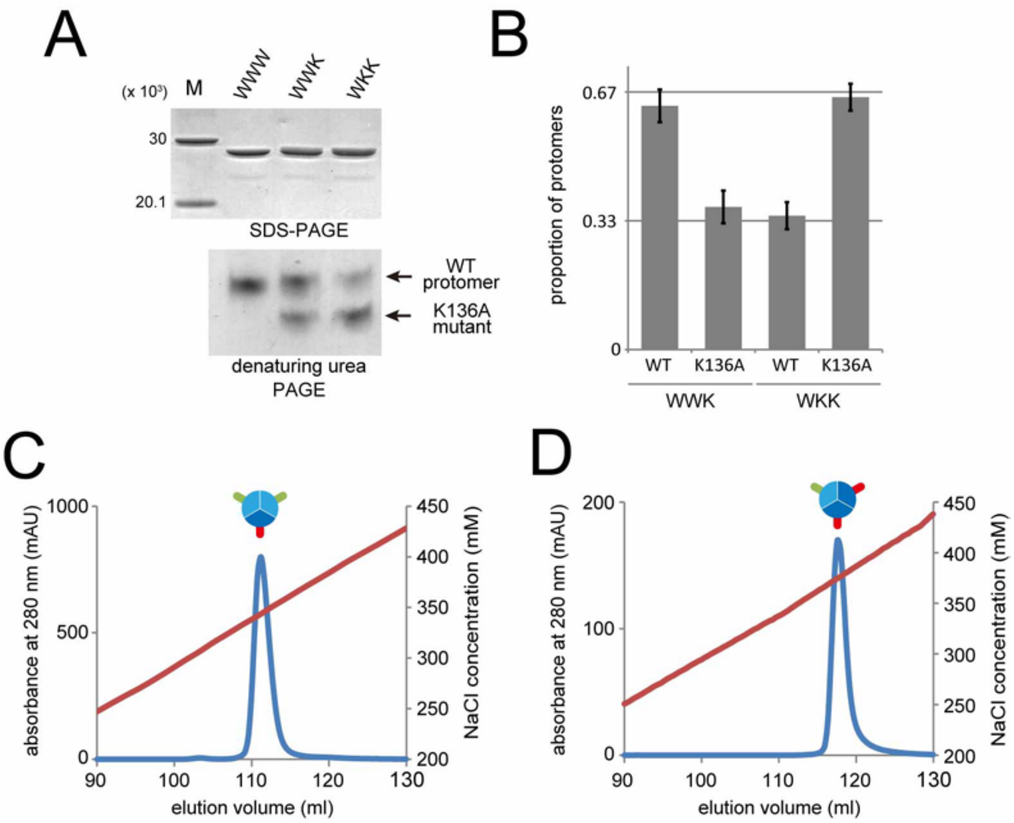

Supplementary Figure 7. Preparation of the PhoExo I heterotrimers. (A) 15% SDS-PAGE and denaturing 10% PAGE of the wild-type trimer (WWW) and the heterotrimers (WWK and WKK). (B) Semi-quantification of the denaturing 10% PAGE gel. Values are mean ± SD (*n* = 3). (C), (D) Stability of the heterotrimers. The heterotrimers were incubated at 65°C for 30 min and were analyzed using an anion exchange chromatography (MonoQ) column. The WWK heterotrimer (C) and the WKK heterotrimer (D) were eluted at 340 and 375 mM NaCl, respectively, as single peaks.

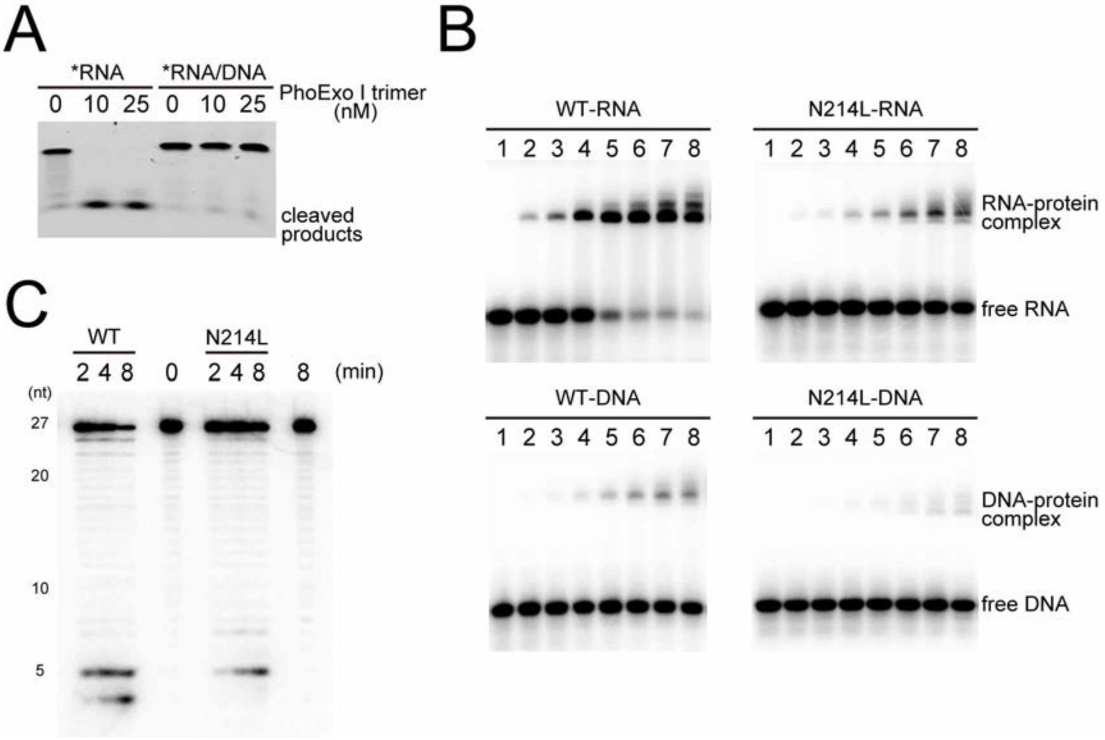

Supplementary Figure 8. Properties of the wild-type PhoExo I and the N214L mutant against RNA substrates. (A) The 3’-5’ exonuclease activities of PhoExo I against the RNA and the RNA/DNA hybrid. The 5′-fluorescein-labeled RNA substrates, marked with asterisks, are indicated at the top of the panel. The products were separated by 15% native-PAGE. (B) Electrophoresis mobility shift assays of the wild-type PhoExo I and the N214L mutant. The 5′-^{32}P-labeled RNA or DNA (5 nM) was incubated with various concentrations (0, 25, 50, 100, 200, 400, 800 and 1600 nM as a trimer) (lanes 1-8) of the wild-type PhoExo I or the N214L mutant. The free probes and the protein-RNA or protein-DNA complex were separated by 7.5% native-PAGE. (C) The 3′-5′ exonuclease activities of the wild-type PhoExo I and the N214L mutant against the 5′-^{32}P-labeled RNA substrate. The reaction times are indicated at the top of the panel. The products were separated through a denaturing 18% polyacrylamide gel.

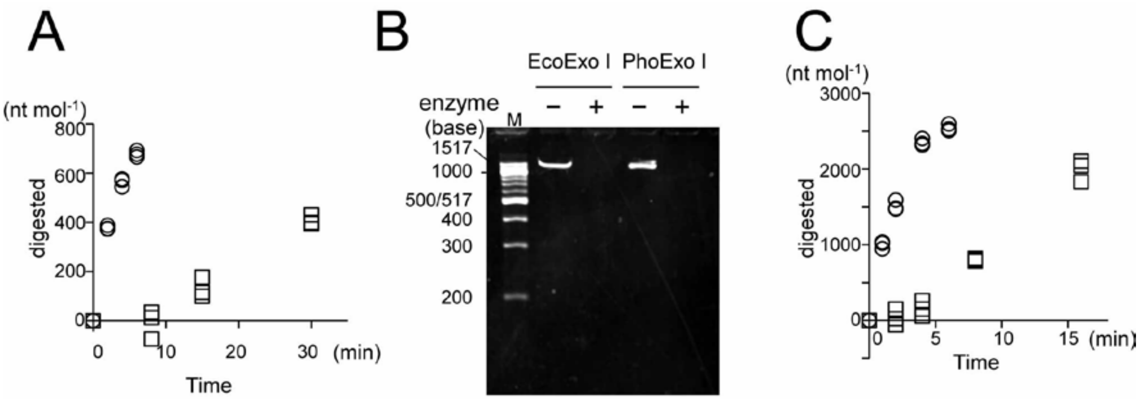

Supplementary Figure 9. Quantitative analyses of PhoExo I nuclease for DNA and RNA. (A) The oligonucleotides of DNA and RNA (30 mer) with the same sequence except T or U were used as substrates and the reactions were performed in the reaction mixture containing 25 mM Tris HCl (pH 8.0), 50 mM NaCl, 5 mM $MgCl_2$, 0.1 mg/ml BSA, 1 μM (30 μM nucleotides) substrates, and 25 nM PhoExo I (as a trimer) at 65°C. The digestion products were plotted for each time, and the initial, linear portion of the curve was fit to determine the slope. Open square, DNA; open circular, RNA. (B) As a long DNA substrate, poly-dT was used for the cleavage assay. In 10 μl of the same reaction mixture as A, 50 ng of poly-dT was digested by EcoExo I (5 units) at 37°C or PhoExo I (5 pmol) at 65°C for 30 min. The reaction mixtures were loaded onto 8 M urea 6% PAGE and the gel was stained by SYBR-Gold. The size marker DNA (New England Biolabs) was loaded on the left lane indicated M. The size of each band is shown on the left side. (C) The reaction rates for long DNA and RNA substrates were obtained by the same experiments as shown in panel A. Poly-dT DNA and RNA Marker (SIGMA) were used (25 ng/μl) and reactions were performed with 25 nM PhoExoI at 85˚C to avoid the formation of the secondary structure of RNA. Open square, DNA; open circular, RNA.

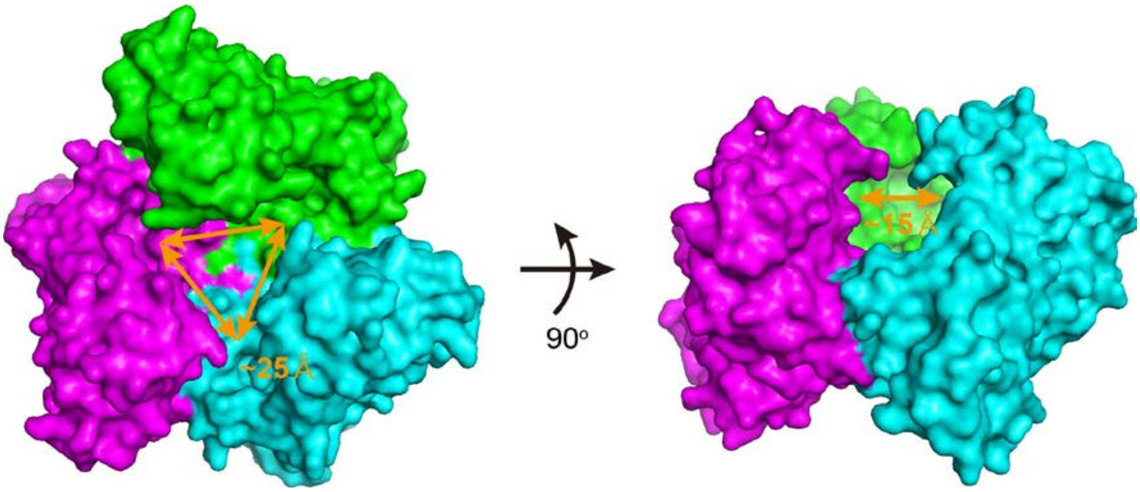

Supplementary Figure 10. Gateway to the active sites of the PhoExo I trimer. The PhoExo I protomers are shown as surface models colored green, magenta, and cyan.

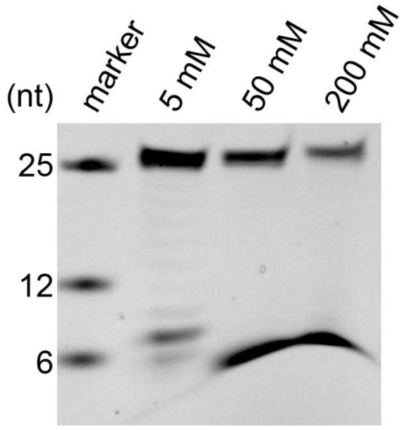

Supplementary Figure 11. Mg^{2+} concentration-dependent 3’-5’ exonuclease activity of PhoExo I. A total of 100 nM of the fluorescein-labeled poly-dT and 16.7 nM of the PhoExo I trimer were mixed in buffers containing 5, 50, and 200 mM Mg^{2+} and were incubated at 50°C for 10 min. The products were separated through a denaturing 18% polyacrylamide gel. The 5’-fluorescein-labeled poly-dTs (6-nt, 12-nt, and 25-nt) were loaded on the gel to provide markers.

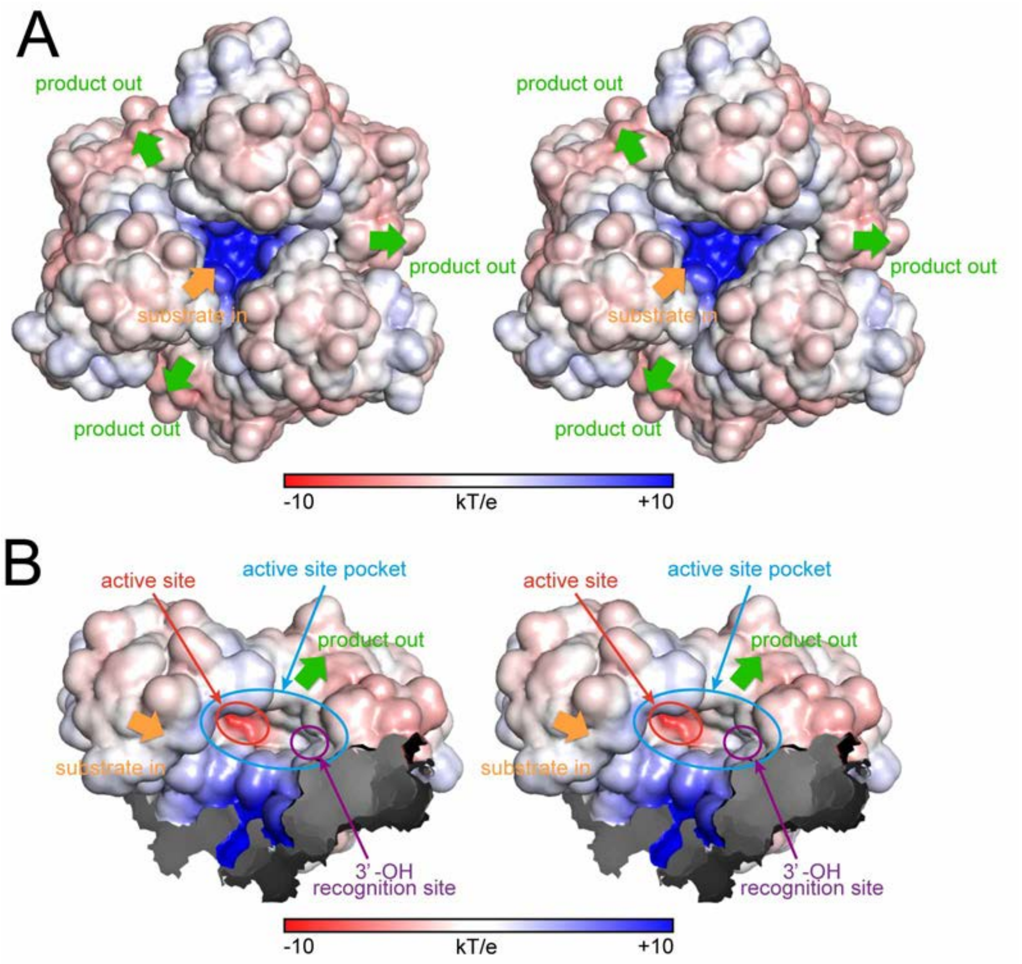

Supplementary Figure 12. Stereo views of the electrostatic potential of PhoExo I. (A) Wall-eyed stereo image of Figure 6A. The ±10 kT/e electrostatic potential is plotted on the solvent-accessible surface of the PhoExo I trimer. (B) Wall-eyed stereo image of Figure 6B. The ±10 kT/e electrostatic potential is plotted on the solvent-accessible surface of the PhoExo I protomer. The active site, the active site pocket, and the 3'-OH recognition site are indicated by red, cyan, and purple circles, respectively.

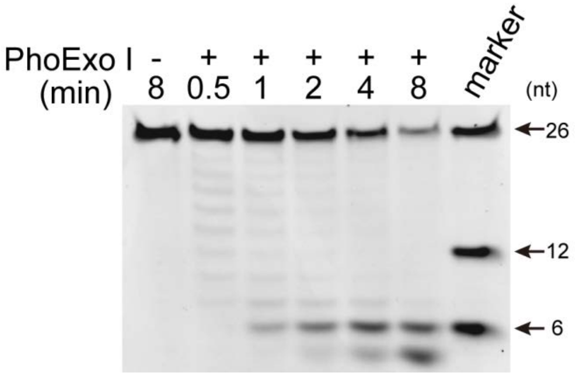

Supplementary Figure 13. Time course analysis of the 3’-5’ exonuclease activity of PhoExo I using 26-nt poly-dT as a substrate. The 5’-fluorescein-labeled poly-dT (26-nt, 100 nM) was incubated with PhoExo I (33.3 nM as a trimer) for the indicated times. The products were separated through a denaturing 18% polyacrylamide gel. The 5’-fluorescein-labeled poly-dTs (6-nt, 12-nt, and 26-nt) were loaded on the gel to provide markers.
